# Sea Level Rise Damages on Ecosystem Quality—A Life Cycle Impact Pathway

**DOI:** 10.1021/acs.est.6c04798

**Published:** 2026-09-28

**Authors:** Nele Teutloff, Martin Dorber, Peter K. R. Holzapfel, Matthias Finkbeiner, Alexis Laurent

**Affiliations:** † Chair of Sustainable Engineering, Institute of Environmental Technology, 26524 Technische Universität Berlin, Berlin 10623, Germany; ‡ Section for Quantitative Sustainability Assessment, Department of Environmental and Resource Engineering, 5205 Technical University of Denmark, Lyngby 2800, Denmark; § Department of Energy and Process Engineering, 8018 Norwegian University of Science and Technology, Trondheim, Trøndelag NO-7491, Norway

**Keywords:** climate change, flood model, characterization factor, biodiversity, species-area relationship, life cycle assessment

## Abstract

Climate change causes species extinction not only by the direct increase in surface temperatures but also by indirect mechanisms, like sea level rise (SLR). These indirect mechanisms are rarely integrated into environmental impact assessment methods, such as life cycle assessment (LCA). In this study, we address this gap by outlining a new impact pathway within the life cycle impact assessment framework that links greenhouse gas (GHG) emissions to ecosystem damages via climate change-induced SLR for 244 different GHGs. We use the latest global digital terrain model with corrected referencing between sea level and coastal elevation for a new globally differentiated and open-source flood model predicting the areas flooded by SLR with a spatial resolution of 1 arc-second (∼30 m). The effects on ecosystems are quantified by computing the potentially disappeared fraction of species caused by flooding in coastal ecoregions worldwide for five terrestrial taxa. A spatial analysis of the results shows that vascular plants and reptiles face the highest extinction risks from SLR, with regional hot spots identified in Central America and across many islands of Southeast Asia. We recommend integrating the developed impact pathway into the LCA framework to better capture impacts to species-rich and particularly vulnerable coastal ecoregions.

## Graphical abstract

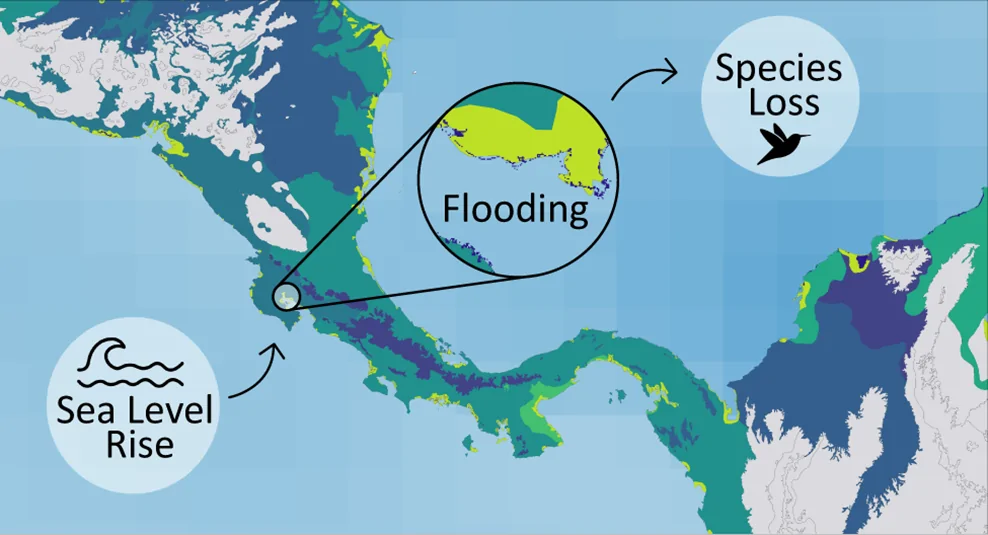

## Introduction

Life cycle assessment (LCA) is a widely used tool to quantify environmental impacts of man-made products and systems from a life cycle perspective (i.e., from raw material extraction, through production and use, up to recycling and final disposal).^1^ As part of LCA, life cycle impact assessment (LCIA) translates emissions and resource use into potential environmental impacts across various impact categories, such as climate change and water use. This is done using characterization factors (CFs), which link elementary flows, such as GHG emissions and water use, to their effects by modeling impact pathways. At the damage level, the entire impact pathway is captured, allowing aggregation of damages to three areas of protection (AoPs): ecosystem quality, human health, and natural resources.^1,2^

Damage to ecosystem quality, capturing biodiversity loss, is calculated by the potentially disappeared fraction of species (PDF). Biodiversity loss is receiving increasing attention as the severity of threats it faces grows more severe and the importance of its services to society becomes widely recognized. As of 2024, biodiversity has declined by up to 6% per decade as a result of human activities, and a sixth mass extinction is already projected.^3,4^ A major driver is anthropogenic climate change, which directly contributes to species extinction and increasingly amplifies the effects of other biodiversity stressors.^5–7^

Although ecosystem quality is highly relevant, current LCIA methods, such as ReCiPe, IMPACT World+, and LC-Impact, cover only a small part of impact pathways going from climate change to ecosystem quality. These are primarily surface temperature rise impacts on terrestrial species, effects from changes in water discharge on freshwater species, and potential loss of marine species from ocean acidification.^8–10^ Other indirect impacts of climate change, e.g., increasing water temperature,^11,12^ alterations in water salinity,^13,14^ and the spread of invasive alien species,^15^ have been studied, and corresponding CFs were developed. However, these impacts are not always explicitly linked to the climate change impact pathway, often face several limitations such as limited species and spatial coverage, and have not yet been integrated into mainstream LCIA methods.

One important but still missing impact pathway for ecosystem quality is climate-change-induced sea level rise (SLR). According to the Intergovernmental Panel on Climate Change (IPCC), global mean SLR is predicted to reach between 0.28 and 1 m by 2100, depending on the shared socioeconomic pathway (SSP).^16^ This leads to a wide range of effects on ecosystems, including coastal erosion, habitat loss due to land submergence, and increasing flood risks.^16,17^ The latest study on SLR indicates that risks have so far been consistently underestimated due to absent or incorrect alignment between sea level and elevation data and, therefore, need to be reassessed.^18^ However, even previous assessments project that risks to coastal ecosystems are very likely to increase 10-fold by 2100 in the absence of adaptation, leading to widespread loss across low-lying and erodible coasts.^16,17^ These ecosystems support a unique and diverse range of species, often at risk of large-scale habitat loss due to flooding caused by SLR. To date, no LCIA methodology accounts for the damage induced by SLR.

In this research, we address this gap by developing an impact pathway linking GHG emissions to SLR and its damages on ecosystem quality. We focus on SLR-induced flooding of terrestrial coastal ecoregions, for which we developed a globally differentiated flood model using the novel digital terrain model (DTM) with corrected alignment between sea level and coastal elevation with a high spatial resolution of 1 arc-second. The flood model further applies the latest IPCC SLR projections for five SSPs and computes flood-induced habitat loss in coastal ecoregions. The impact pathway further links habitat loss to ecosystem damages for five terrestrial taxa—amphibians, birds, mammals, reptiles, and vascular plants—through the countryside species–area relationship (c-SAR). Finally, we calculate spatially differentiated CFs that enable the quantification of ecosystem damages at a global scale with a high coverage in terms of terrestrial species and GHGs and which can be easily integrated into the existing LCIA framework.

## Materials and Methods

### Framework

The LCIA characterization framework, used to model impact pathways, can consist of four main factors that form the CFs of substance emission *i*.^10^ The fate factor (FF) addresses the distribution and transformation of a given substance following its emission, while the exposure factor (XF) typically analyzes the level of contact between organisms and the substance after its distribution and degradation in the environment. The effect factor (EF) describes the observed adverse effects of the organisms. Vulnerability factors (VFs), also termed severity or damage factors, capture the severity of the adverse effects on the organisms or, in the case of ecosystem quality, translate species loss from local or regional to global.^10,19^

In this study, we used the framework to develop an impact pathway for climate change-induced SLR as shown in Figure 1, leading to eq 1,

1 $$CF_{i,j,g}=\overset{FF_{i}}{\overset{︷}{\frac{\Delta Temp_{\mathrm{gl},\mathrm{cum}}}{\Delta GHG_{i}}}}\cdot \overset{XF_{j}}{\overset{︷}{\frac{\Delta SLR_{loc}}{\Delta Temp_{\mathrm{gl},\mathrm{cum}}}\cdot \frac{\Delta AreaF_{j}}{\Delta SLR_{loc}}}}\cdot \overset{EF_{j,g}}{\overset{︷}{\frac{\Delta EQ_{j,g}}{\Delta AreaF_{j}}}}\cdot \overset{VF_{j,g}}{\overset{︷}{GEP_{j,g}}}$$

with the corresponding units as in eq 2,

2 $$CF_{i,g}=\overset{FF_{i}}{\overset{︷}{\frac{K\cdot yr}{kg}}}\cdot \overset{XF_{j}}{\overset{︷}{\frac{m}{K\cdot yr}\cdot \frac{m^{2}\cdot yr}{m}}}\cdot \overset{EF_{j,g}}{\overset{︷}{\frac{PDF}{m^{2}}}}\cdot \overset{VF_{j,g}}{︷}=\frac{PDF\cdot yr}{kg}$$

**Figure 1:**
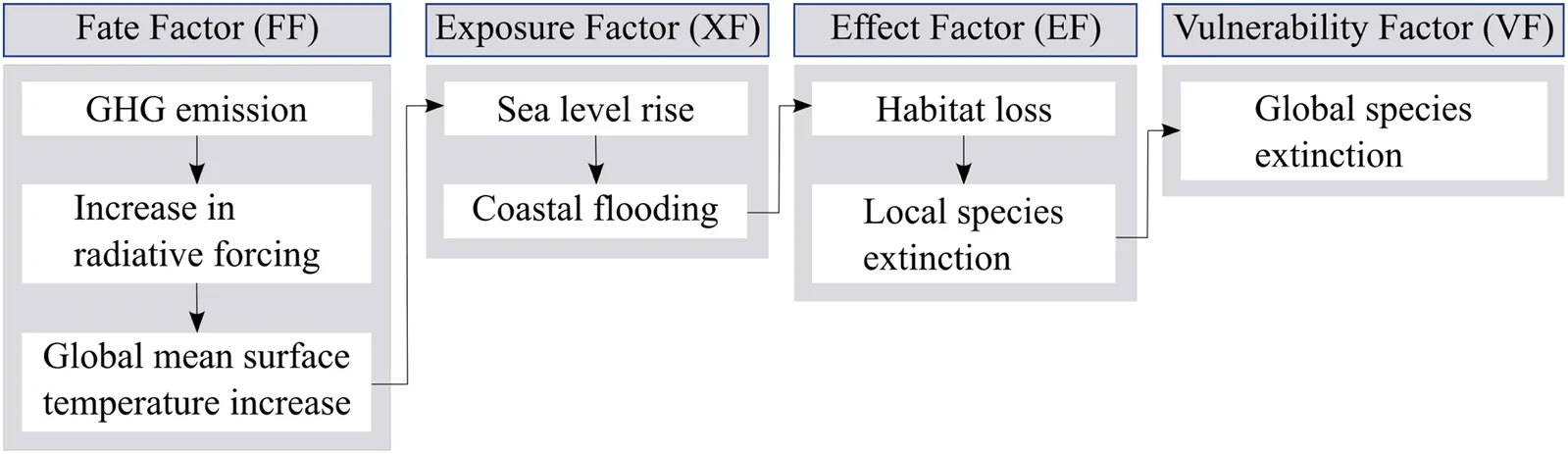
Overview of the impact pathway for climate change-induced SLR with details about FF, XF, EF, and VF.

Here, FF_*i*_ represents the cumulative global surface temperature increase over 85 years (ΔTemp_gl,cum_) in Kelvin·year caused per kilogram of emitted GHG_*i*_ [kg]. We subdivided XF_*j*_ into two parts that together model the loss of terrestrial area in each ecoregion due to SLR. For the first part, we define ΔTemp_gl,cum_ as the cumulative global surface temperature change over 85 years [K·yr] and ΔSLR_loc_ as the local change in mean sea level [height in m] due to the cumulative temperature increase. In the second part, we quantify the extent of area ΔAreaF_*j*_ continuously flooded over time [m^2^·yr] in an ecoregion *j* for as long as local SLR persists.

The EF_*j*,*g*_ covers the decrease in local ecosystem quality ΔEQ_*j*,*g*_ due to a habitat loss by flooding for taxon *g* in the affected ecoregion *j*, measured as the potentially disappeared fraction of species (PDF). Note that PDF does not represent the actual number of species going extinct, but the fraction of species being potentially lost. To translate the potential regional species loss to a global species loss, we use global extinction probabilities (GEP_*j*,*g*_) as a VF_*j*,*g*_. The GEP are scaling factors that quantify the likelihood of global extinctions based on local species loss and are derived from, among others, species range sizes and global conservation status.^20^ The resulting CF_*i,j,g*_ is the globally differentiated and taxon-specific PDF·yr/kg for ecoregion *j* and taxon *g*. As the FF_*i*_ is integrated over 85 years and XF and EF are evaluated for the end of this time horizon, the resulting CF_*i,j,g*_ describes the effect of SLR at this specific point in time, here after 85 years. We further aggregate CF_*i,j,g*_ across ecoregions and taxa (amphibians, birds, mammals, reptiles, and vascular plants, giving each group the same weight). The aggregation leads to a global CF_*i*_ that is taxon-generic and GHG-specific, as in eq 3 with *N* being the number of taxa.

3 $$CF_{i}=\frac{\underset{j,g}{\sum }CF_{i,j,g}}{N}$$

In the following sections, we provide details on the development of each individual factor of the new impact pathway (Figure 1) and eq 1.

### Fate Factor

The FF describes the transport, transformation, and distribution of GHGs emitted to the atmosphere, as well as their contribution to climate change through increased radiative forcing. Here, we use the time-integrated Absolute Global Temperature Potential (iAGTP) to express the cumulative climate change over 85 years. This climate metric quantifies the cumulative global mean surface temperature increase [K·yr·kg^–1^] caused by a GHG pulse emission.^21^ We chose an 85-year time horizon to ensure that the integration period for temperature increase is consistent with the time frame used in the SLR projections, which extend from the reference period (1995–2014) to 2100.

The iAGTP was selected because it directly links GHG emissions to long-term climate effects such as SLR, where a continuous temperature increase is the key driver of main SLR mechanisms such as thermal expansion and ice sheet melting. Moreover, SLR models rely on temperature trajectories as input, making GTP the most direct and consistent metric to link GHG emission to SLR. This approach has been applied in previous studies assessing climate change damages^11,22,23^ and is recommended by the UNEP-SETAC Life Cycle Initiative for long-term climate-change modeling.^24,25^

We computed the IAGTP for 244 GHGs based on the IPCC’s Sixth Assessment Report (AR6) with the most recent parameter values from Zieger et al. (2025).^26,27^ For GHGs for which no updated values were available in AR6, values were taken from IPCC’s Fifths Assessment Report (AR5).^21,28^ Details on the calculation and applied data are summarized in the (Supporting Information SI).

### Exposure Factor: Climate Change-Induced Sea Level Rise

The XF is defined as climate-change-induced SLR resulting in coastal flooding. For practical modeling reasons, we divided the XF into two parts. The first part represents the climate-change-induced SLR. Sea level has changed constantly throughout history; however, climate change is expected to cause additional SLR in the future through several mechanisms, such as thermal expansion and ice sheet melting.^16^ Such processes have long response times, up to centuries, and are additionally nonlinear, depending on both the period over which temperature increases and the specific climate scenario.^16,29^ The same temperature change by 2100 can therefore lead to different SLRs depending on the temperature trajectory during the time horizon (e.g., abrupt versus gradual warming). It is therefore necessary to define a point in time and use the cumulative temperature change of a specific climate scenario when working with the SLR. In this study, we use regional relative sea level projections for 2100 with a reference period of 1995–2014. For the climate scenario, we selected five SSPs to account for the uncertainty of the future climate and SLR scenarios. Consequently, the XF is scenario-dependent, unlike the FF, which is scenario-independent, as they are derived from a single pulse emission.

The SLR is modeled by CMIP6, a project of the World Climate Research Program, available on Zenodo and the IPCC WGI Interactive Atlas, with a spatial resolution of 1°.^16,30–32^ To obtain the SLR per temperature increase, we divided the regional relative SLR by the cumulative surface temperature change by 2100 under each SSP obtained from IPCC’s AR6.^33^ As both FF and XF have a specific time horizon, the resulting CF describes the effect of SLR at this specific point in time, here after 85 years.

### Exposure Factor: Sea Level Rise-Related Flooding

The second part of the XF represents the SLR-induced flooding of terrestrial areas within ecoregions connected to the sea. To quantify this, we developed a flood model based on the widely used bathtub approach, assuming that all areas below the projected sea level are inundated.^34^ Areas below the sea level but not hydraulically connected to the ocean were excluded by integrating hydrological connectivity. This is particularly relevant for coastal regions, as coastal floods only affect bordering land zones.^35^ However, inland flood risks due to rising groundwater levels are thereby excluded.

The model uses the novel converted DeltaDTM with a corrected reference to the latest coastal sea-level height as input for terrain elevation data.^18,36^ DeltaDTM provides millimeter-scale accuracy (vertical mean absolute error of 0.43 m) at a spatial resolution of 1 arc-second (∼30 m).^36^ Furthermore, we apply the latest SLR projections from CMIP6 climate models for the five SSPs.^16,30,31^ Antarctica was excluded from the analysis because DeltaDTM does not cover this region and local climatic conditions make SLR impacts negligible for the scope of this study.

We further combined the results of the flood model with terrestrial ecoregions and calculated the inundated area per ecoregion both as an absolute value in km^2^ and relative to the total ecoregion area in percent. We assume that inundated areas are lost for all terrestrial species of the selected taxa and that inundation persists over time as long as SLR does not decrease. With that, it aligns with the definition of land occupation by Scholz et al. (2007),^37^ in which land occupation has both a spatial and temporal dimension and is generally quantified as area × time (m^2^·year). The unit in the XF is therefore m^2^·yr per m of sea level increase accounting for the duration of inundation. The exact timing of inundation, however, is not explicitly accounted for in the model, as SLR flooding is assessed for a single point in time (2100). Furthermore, future adaptation measures to SLR, such as coastal protections, dike construction, or sediment management, are excluded in the model due to practical modeling reasons. Terrestrial ecoregions are defined as ecologically homogeneous regions classified based on biodiversity, environmental properties, climatic conditions, and vegetation.^38^ The ecoregion boundaries follow the proposal by Pfister et al. (2020) for a harmonized regionalization in LCIA^39^ and are obtained from WWF’s Terrestrial Ecoregions of the World dataset.^38,40^

The code for the developed flood model is made publicly available on GitLab.

### Effect Factor

We define the EF as the decrease in ecosystem quality within an ecoregion due to the habitat loss from flooding. This decrease is expressed as the potential relative species loss (RSL) measured in PDF, which is currently the most widely used indicator of ecosystem quality in LCA^2^ and is recommended by the Life Cycle Initiative.^2^ At this point, it is important to note that RSL measures regional species loss, not global species extinction.

To quantify the RSL, we adapted the species–area relationship (SAR), which gives a direct connection between species loss and area decrease,^41^ to the case of SLR. In this case, habitat area shrinks from its original extent (*A*_org_) by the area being flooded (*A*_flooded_), as expressed in eq 4,

4 $$\mathrm{RSL}=\frac{S_{\mathrm{lost}}}{S_{\mathrm{org}}}=1-{\left(\frac{A_{\mathrm{org}}-A_{\mathrm{flooded}}}{A_{\mathrm{org}}}\right)}^{z}$$

where *S*_lost_ represents the expected number of species lost and *S*_org_ is the original number of species. The SAR, thus, reflects the decline in species richness due to the decrease of available habitat, with *z* (dimensionless) representing the slope of this relationship in log–log space.^42^ This general SAR formulation can be further refined by incorporating land cover types and species affinities toward those land cover types, resulting in the countryside species–area relationship (c-SAR) first used in LCIA by Chaudhary et al. (2015).^43^ Integrating the c-SAR into eq 4 and additionally accounting for intensity levels of land use types, the EF is calculated as in eq 5.

5 $${\mathrm{EF}}_{j,g}={\mathrm{RSL}}_{j,g}=1-{\left(\frac{{\left(\sum_{i,m}h_{i,j,g,m}\times A_{i,j,m}\right)}_{\mathrm{org}}-{\left(\sum_{i,m}h_{i,j,g,m}\times A_{i,j,m}\right)}_{\mathrm{flooded}}}{{\left(\sum_{i,m}h_{i,j,g,m}\times A_{i,j,m}\right)}_{\mathrm{org}}}\right)}^{z_{j,g}}$$

*A*_*i,j,m*_ [km^2^] is the area of land cover type *i* with an intensity level *m* in ecoregion *j*, and *h*_*i,j,g,m*_ is the affinity of taxon *g* to land cover type *i* with an intensity level *m* in ecoregion *j*. *z*, defined as the slope of the SAR, is specific to ecoregion *j* and taxon *g*.^42^ The numerator in eq 5 thus represents the sum of flooded land cover areas weighted by species affinity, while the denominator represents the total weighted land cover area prior to flooding.

Although SLR-induced flooding will occur under future conditions with potentially changed land cover patterns and species distributions, we selected the current (2024) state as the reference condition (*A*_org_). We did not select a natural reference state because our aim was, first, to estimate actual species loss under current conditions and, second, to ensure that the EFs remain additive to other biodiversity stressors such as land use. We also did not adjust the reference state over time, as future land cover patterns and species distributions are highly uncertain, and corresponding projections remain limited. However, we acknowledge that the choice of the reference state affects the results and introduces additional uncertainty. Under the current conditions, species are assumed to be homogeneously distributed within ecoregions and land cover types.

This approach is similar to that of Dorber et al. (2020),^44^ who modeled land inundation as a case of land use change. However, for SLR, the inundated areas are spatially explicit and known, which allowed us to simplify the calculations and apply the most recent land cover and species affinity data. The more recent species–habitat relationship by Scherer et al. (2023)^45^ incorporating habitat fragmentation, was not applied here, as SLR occurs primarily along coastal boundaries, where fragmentation effects are assumed to be negligible.

We assessed species loss for five terrestrial taxa—amphibians, birds, mammals, reptiles, and vascular plants—and across all affected ecoregions with available data for species affinities, z parameters, and land cover types. Data were obtained from existing literature sources^45^ (background dataset acquired from authors). When *h*-values for natural habitat were missing, a value of 1 was assigned to be consistent with the classical SAR in eq 4. Only habitat losses due to inundation were considered. Although local sea-level decreases are projected in some regions, the resulting habitat gains were excluded because LCIA generally focuses on negative impacts, and the ecological value of newly exposed land is uncertain.

### Vulnerability Factor

Distinguishing between regional and global species extinction is critical, as species lost regionally may be reestablished, whereas globally extinct species are irreversibly lost. To convert regional biodiversity losses to a global level, we used global extinction probabilities (GEPs), which are scaling factors that estimate the likelihood of global extinctions if species disappear locally.^46^ We applied the most recently updated GEP values provided by Verones et al. (2024) by multiplying the EFs with the corresponding GEP for each taxon group and ecoregion.^47^

The GEPs range from 0 to 1 and are derived from species range sizes, global conservation status (IUCN), occurrence certainty, and species richness.^20^ These scaling factors cover more than 98 000 species across 20 taxa in marine, terrestrial, and freshwater ecosystems. For terrestrial ecosystems, factors are available for vascular plants, mammals, reptiles, amphibians, and birds in aggregated form for relevant spatial units, such as ecoregions.^20^

### Uncertainty Assessment

We quantitatively assessed the uncertainty of the developed impact pathway regarding the variability across different future climate-change scenarios. We modeled both parts of the XF with the five SSPs for which SLR projections were available, namely, SSP1-1.9, SSP1-2.6, SSP2-4.5, SSP3-7.0, and SSP5-8.5. This results in distinct cumulative temperature increases in XF part one and different SLR projections in XF part two. On the basis of the resulting variations in XFs, we derived SSP-specific CFs. In addition, within each SSP, the projected temperature increase by 2100 is given with an uncertainty range represented by the mean, 5th, and 95th percentile values. We applied this range in XF part one and further incorporated it into the CFs. Thereby, we calculated CFs for each SSP and, within each SSP, for the mean, 5th, and 95th percentile probabilities of temperature increase. As the default scenario and central result, we chose SSP2-4.5 since it is assessed as most likely based on current policies.^48^ Besides future climate change, uncertainties also arise from parameters used in the AGTP and RSL calculations, such as species affinities, as well as from the GEP applied. Additionally, the use of terrain elevation data introduces uncertainties that propagate to the flood results. These uncertainties are not captured in the quantitative uncertainty assessment but are addressed qualitatively, where relevant.

## Results and Discussion

First, we present and analyze the results of the XF and EF, which constitute the key methodological advances of the work, followed by the resulting CF as the final outcome. Results related to the FF and the applied values for the VF are provided in the SI. Unless otherwise stated, we show results for SSP2-4.5 as the mean, with the uncertainty arising from the different SSPs expressed as ${\mathrm{mean}}_{-\Delta_{\min}}^{+\Delta_{\max}}$ , where the superscript and subscript indicate deviations of the mean to the maximum and minimum values.

### Exposure Factors and Flood Model Validation

Overall, between 459 and 460 of the world’s 867 terrestrial ecoregions are projected to be affected by SLR, and an area of 249,301–420,638 km^2^ will be lost due to SLR flooding, depending on the SSP. Although more than half of all ecoregions will be impacted, most experience low inundation rates, losing only a small proportion of their total area, as shown in Figure 2B. In Figure 2A, flooding is shown exemplarily for northern Australia, where substantial inundation occurs along the coastline. However, it remains small relative to the total ecoregion area. The relative loss increases for ecoregions with small total areas where flooding can inundate more than 40% of their total surface (Figure 2). This pattern aligns with the fact that many ecoregions have only narrow coastal zones with large inland areas, while a comparatively small number of island ecoregions are far more exposed to large-scale flooding.

**Figure 2:**
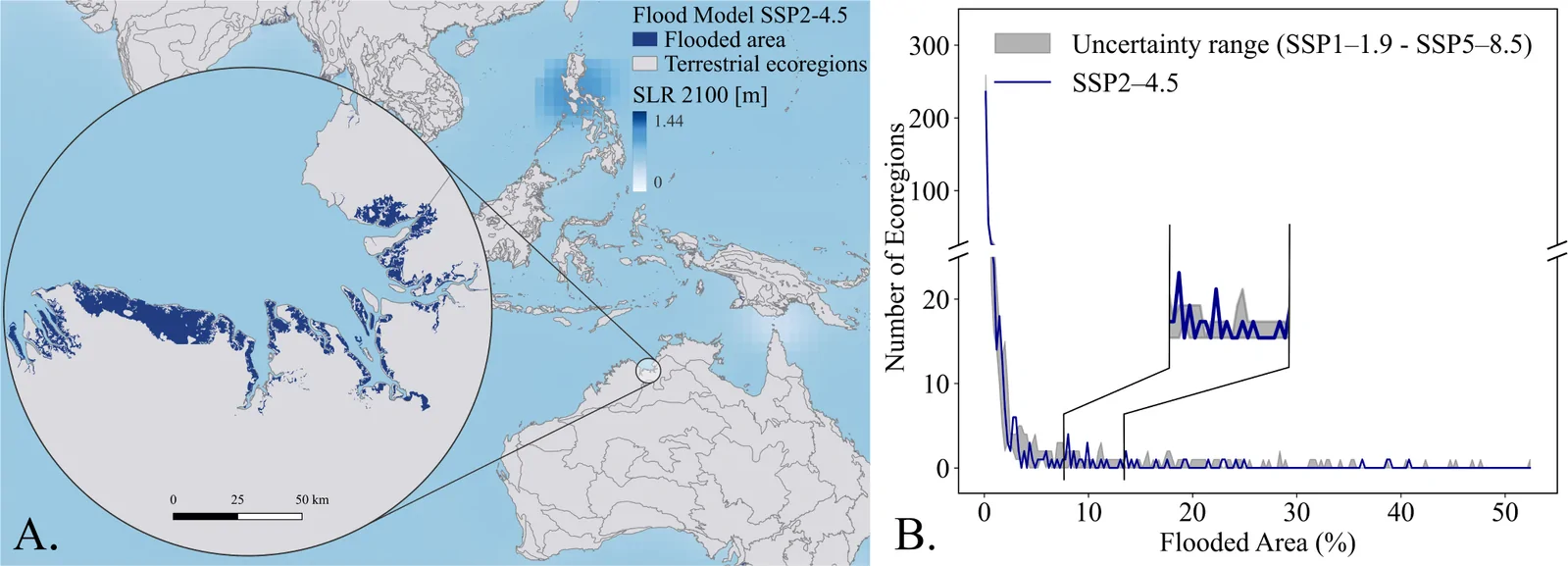
Flood model results for SSP2-4.5 by 2100: A. Local SLR and flooded areas for north Australia (flooded area in dark blue, SLR ranges from white to dark blue, and ecoregions in light gray). B. Distribution of flooded area (%) across ecoregions for SSP2-4.5 in blue, with the uncertainty range across other SSPs in gray.

The ecoregions with the largest absolute flooded areas are the Atlantic mixed forests (western Europe: 17,387 km^2^ flooded) and the Western Gulf coastal grasslands (Texas, USA: 17,336 km^2^ flooded), consistent with low elevation levels at western European coasts and regional SLR projections of up to 1.44 m along the eastern coast of North America.^16^ The Mesoamerican Gulf-Caribbean mangroves and Indochina mangroves show the highest relative area loss across SSPs ( $39.0_{-3.4}^{+5.8}\%$ and $38.7_{-13.9}^{+13.8}\%$ ), matching the expectation that small ecoregions with low-lying islands face the greatest risk of proportional area losses. We summarize the minimum, median, and maximum flooded areas expressed in both km^2^ and percent for all SSPs, along with the corresponding ecoregions, in Tables S4 and S5.

Overall, the uncertainty range across SSPs is small to moderate, indicating that the results are robust across climate scenarios, while mitigation toward lower GHG emission pathways remains impactful. The robustness suggests that spatial hot spots of future habitat loss can already be reliably identified now, enabling targeted conservation and adaptation planning in a timely manner. Furthermore, the results are not specific to biodiversity loss and can therefore also support assessments of risks to human health and socioeconomic assets.

The flood model performance was evaluated against two independent flood models of varying spatial resolutions and different elevation models for the region of Zealand, Denmark. Our flood results obtained using the corrected DeltaDTM lie between those of the two comparison models but are closer to the higher-resolution national model. The observed differences are consistent with variations in flood model resolution and underlying elevation data, with a higher resolution and improved elevation data leading to lower flood extents in this specific case. Overall, the similarity in the flood patterns supports the robustness of the developed model. Detailed results can be found in the SI.

It is important to note that the year 2100 represents a relatively short time horizon to assess the impacts of SLR, as sea levels are expected to continue to rise and threaten coastal areas for centuries due to long-term ocean warming.^16^ Extending the time horizon in future analysis of SLR could therefore provide a more comprehensive assessment, despite higher uncertainties in future climate scenarios. Another concern of future scenarios is adaptation measures, which were excluded from this study. This can lead to an overestimation of impacts, especially in regions with advanced coastal protection infrastructure in place or planned, such as Western Europe (e.g., the Netherlands),^49^ North America^50^ (e.g., the US East Coast),^51^ and Australia^52^—regions of highest projected flooding. Conversely, results for South and Southeast Asia and Small Island Developing States (SIDS) are less affected by this assumption, as adaptation capacity in these regions is considerably lower.^53^

Another limitation concerns freshwater areas, such as river basins discharging into the ocean, which the flood model may partially assign to terrestrial ecoregions because they are classified as such in the underlying ecoregion layer.

### Effect Factor and Spatial Analysis

In total, we calculated 2,754–2,760 EFs per SSP, covering 459–460 coastal ecoregions, 5 taxa, and a taxon-generic group. The EFs are computed using the flood model results and are therefore both time- and SSP-specific. For a spatial analysis of the effects of SLR, we multiplied the projected flooded area under each SSP with the EFs, or EFs and VFs to identify regional and global taxon-specific hot spots at real future risk.

Based on this analysis, we found that the Bahamian-Antillean mangroves show the highest regional as well as global fraction of species loss across taxa. The Bahamian-Antillean mangroves are part of the Bahamas islands, consisting of 3,000 low-lying islands with a maximum elevation of 60 m. They inhabit several endemic bird species, and their mangroves provide important shelter to terrestrial species, leading to high vulnerability toward rising sea levels.^54^ Across all ecoregions, taxon-generic factors yield an average regional PDF of $4.7_{-1.0}^{+1.6}$ E-3 and a global PDF of $2.6_{-0.6}^{+0.8}$ E-6.

Among taxa, vascular plants and reptiles have the highest average factors, followed by amphibians. Birds show the lowest regional species loss ( $3.2_{-0.7}^{+1.1}$ E-3 PDF), while mammals have the lowest global PDF of $1.8_{-0.4}^{+0.6}$ E-6. The results align with our expectation, as plants have the lowest mobility and are expected to be the slowest in shifting their habitat inland. The high values for reptiles align with the CFs developed by Dorber et al. (2020) for inundation of different land use types, where global CFs for reptiles have the highest maximum values for most land use types.^44^ Contrary to our results, birds exhibit the highest values for most regional CFs in their study.

Figure 3 shows the results of potential loss of vascular plant species regionally and globally. Hot spots of regional reptile species loss are projected for including Central America, including the Bahamas islands, where high SLR is projected, as well as for many islands of Southeast Asia. However, regional hot spots do not necessarily lead to hot spots of global species extinction, as is visible when comparing Alaska in Figure 3A and B. In contrast, ecoregions in Central America and Southeast Asia show continuous high values for global species loss, and some ecoregions even become new hot spots, such as those in northwest Australia. A main reason can be the occurrence of endemic or highly threatened species in these regions.

**Figure 3:**
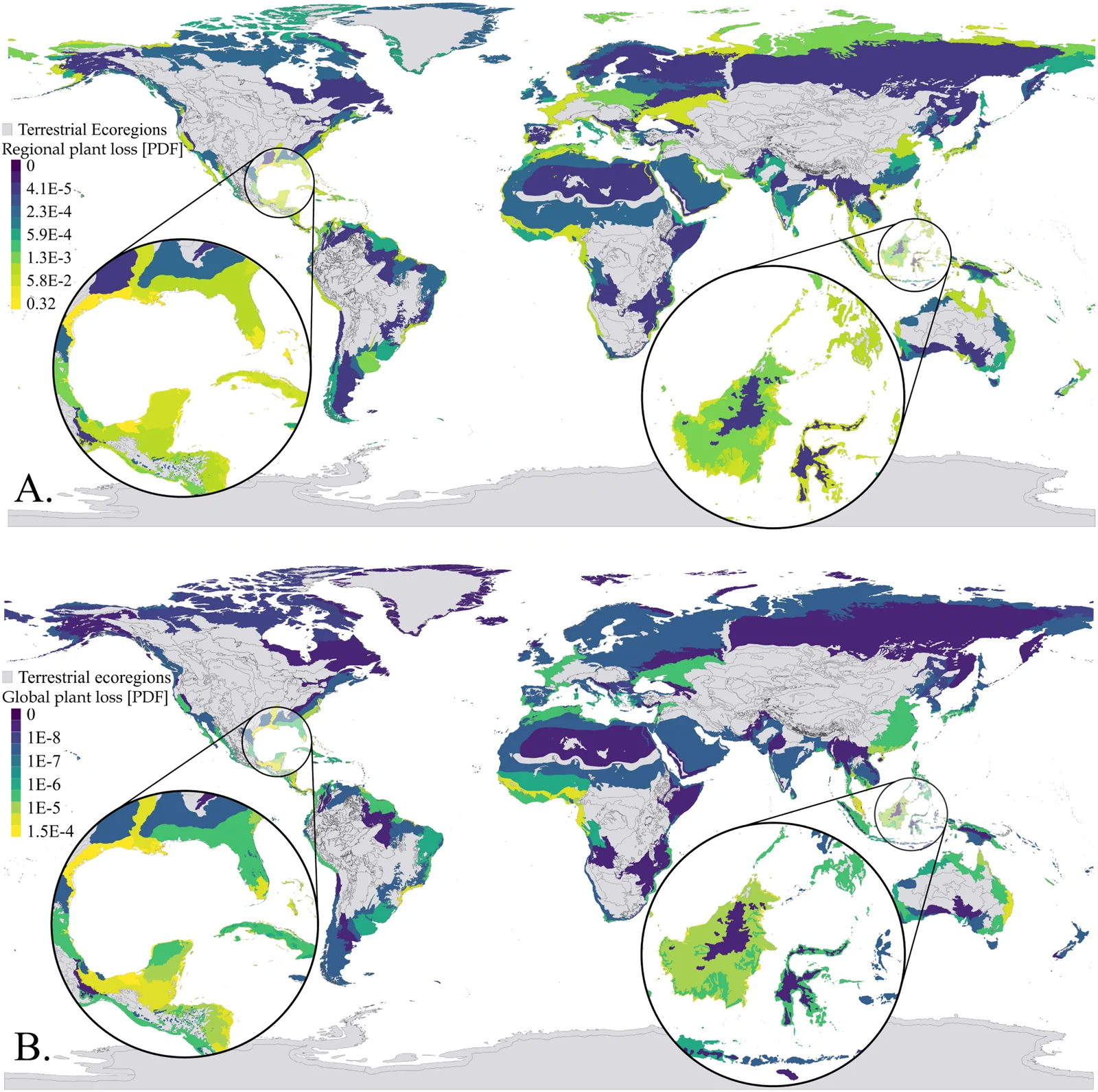
Spatial analysis of ecosystem damages for SSP2-4.5 by 2100: A. Regional effects on vascular plants (SSP2-SLR · EF) and B. Global effects on vascular plants in PDF (SSP2-SLR · EF · VF), with highlighted areas of Central America and Southeast Asia. Ecoregions in gray are not affected by SLR.

Although flooded areas are relatively small, EFs can reach severe levels of up to 32% regional species loss due to two main reasons: 1. Coastal areas are often natural habitats with high species affinity values (*h*), and 2. Many islands host a high proportion of endemic species,^55^ leading to a high global species loss under local flooding in these ecoregions.

For some land cover types in specific ecoregions, missing *h*-values (taxon affinity values) resulted in flooded areas not being accounted for in the corresponding EFs, potentially underestimating impacts. For natural habitats, a value of 1 was assigned to missing *h*-values to maintain consistency with the classical SAR. Detailed results of the spatial analysis for all taxa are available in the SI (Figure S2–Figure S11 and online via Zenodo).

### Characterization Factors

We calculated 1,220 taxon-specific and 244 taxon-generic CFs per SSP for the mean temperature increase and complemented them with CFs for 5th and 95th percentile temperature-increase probabilities. In Table 1, we provide mean CFs under SSP2-4.5 for the seven main GHGs from the Kyoto Protocol. For HFCs and PFCs, the substances with the highest CFs are reported exemplarily to outline the maximal potential impacts. CFs for all 244 GHGs and 5 SSPs are documented in open access in the SI’s respective Excel files (and online via Zenodo).

**Table 1:** Taxon-Specific and Taxon-Generic Global CFs for Seven GHGs Stated by the UNFCCC/Kyoto Protocol^56^ under SSP2-4.5 in PDF·yr/kg with Selected HFC and PFC Substances to Illustrate the HFC and PFC Groups

CF [PDF·yr/kg]	CO_2_	CH_4_	N_2_O	HFC-23^a^	PFC-116^a^	SF_6_	NF_3_
Taxon generic	9.12 × 10^–19^	1.08 × 10^–17^	9.05 × 10^–17^	4.82 × 10^–15^	3.93 × 10^–15^	8.00 × 10^–15^	5.61 × 10^–15^
Amphibians	6.18 × 10^–19^	7.30 × 10^–18^	6.13 × 10^–17^	3.26 × 10^–15^	2.66 × 10^–15^	5.42 × 10^–15^	3.80 × 10^–15^
Birds	6.89 × 10^–19^	8.14 × 10^–18^	6.84 × 10^–17^	3.64 × 10^–15^	2.97 × 10^–15^	6.04 × 10^–15^	4.24 × 10^–15^
Reptiles	1.71 × 10^–18^	2.02 × 10^–17^	1.69 × 10^–16^	9.00 × 10^–15^	7.35 × 10^–15^	1.49 × 10^–14^	1.05 × 10^–14^
Vascular plants	8.88 × 10^–19^	1.05 × 10^–17^	8.81 × 10^–17^	4.69 × 10^–15^	3.83 × 10^–15^	7.78 × 10^–15^	5.46 × 10^–15^
Mammals	6.45 × 10^–19^	7.62 × 10^–18^	6.40 × 10^–17^	3.40 × 10^–15^	2.78 × 10^–15^	5.65 × 10^–15^	3.97 × 10^–15^

a CFs for an example of the respective groups. CF of all substances belonging to the groups of HFC and PFC as well as GHGs not stated by the Kyoto Protocol but included in the study can be found in the SI and online via Zenodo.

Overall, the CFs vary over 8 orders of magnitude across GHGs while remaining consistent in magnitude across taxa. Thus, it is reasonable to apply taxon-generic CFs when no differentiation between taxa is possible. However, it also highlights the importance of differentiating between GHGs and further shows that the FF is numerically the dominant factor in the results. Although the AGTP used for the FF is a scientifically robust climate metric, it is associated with larger uncertainties than other climate matrices further up the cause-effect chain, such as the AGWP.^28,57^ These uncertainties could not be assessed here due to missing uncertainty ranges of AGTP parameters. Given the apparent sensitivity of the CFs to the FF, we recommend evaluating AGTP-related uncertainty in future climate change impact pathways.

Across SSPs, the overall magnitudes of the CFs remain stable, showing the robustness of the results. Yet, the uncertainty ranges are still substantial, which highlights the need to differentiate between climate scenarios and to further include other uncertainty sources when developing climate change impact pathways. This is further illustrated in Figure 4, where selected CFs are shown together with their minimum and maximum values across SSPs and percentiles.

**Figure 4:**
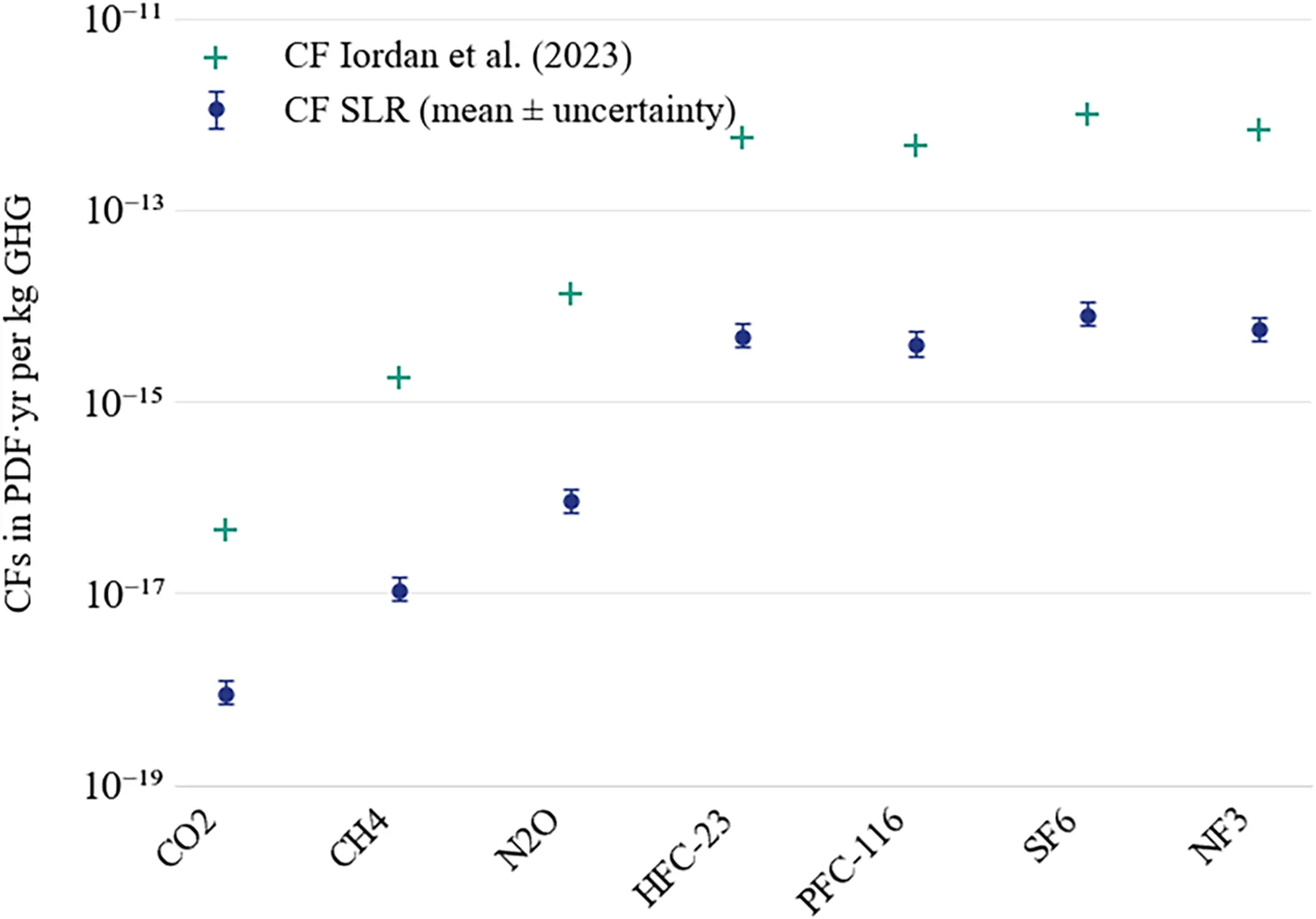
Comparison of CFs for seven GHGs defined by the Kyoto protocol.^56^ CFs for temperature increase in PDF·yr/kg from Iordan et al. (2023)^23^ are presented in light blue crosses, and CFs for SLR (this study) in PDF·yr/kg are visible as dark blue dots with minimum and maximum values.

The calculated CFs are time-specific (by 2100 in this study) with the unit PDF·yr/kg. In the SI FF_EF_CF_to_use.xls file, the key CFs are reported as the mean values for SSP2-4.5, together with the minimum and maximum values across all SSPs and percentiles. In addition, a shapefile containing the spatially differentiated EFs, EF × VF combinations, and CO_2_-specific CFs for SSP2-4.5 is provided for download via Zenodo.

### Relevance of the Newly Developed Impact Pathway

To evaluate the relevance of the developed CFs, we compared our taxon-generic CFs with the most recent taxon-generic CFs for climate change impacts on terrestrial ecosystems from Iordan et al. (2023).^23^ The impact pathway covered in their work captures the direct damages of near-surface temperature increase on terrestrial species while excluding effects from indirect mechanisms such as SLR. The modeling of the FF in their study is similar to ours, as they also employed AGTP values together with projected temperature increases for SSP2-4.5 by 2100. However, while we rely primarily on AR6 ^26^ values for AGTP calculation, Iordan et al. used AR5 data, leading to GHG-specific differences.^23^ Furthermore, only four of the five taxa used in our work are covered in that previous study. For the comparison, we obtained the CFs of Iordan et al. from the LCIA method GLAM in the unit of PDF·yr/kg, making a direct comparison possible.

As can be seen in Figure 4, CFs for SLR are around 2 orders of magnitude smaller than those of surface temperature increase. The smaller impact of SLR is in line with expectations, as SLR affects only coastal ecoregions and primarily along the coastline. In addition, many coastal ecoregions have relatively small coastal areas compared to large inland habitats affected by temperature increase. Thus, when spatially aggregated into global CFs, the impacts of SLR appear relatively small. However, this aggregation disregards the relevance of the SLR impact pathway for specific coastal ecoregions. For example, vulnerable low-lying ecosystems such as the Bahamian–Antillean mangroves can experience potential regional species loss of up to 30%, highlighting the high sensitivity of certain coastal ecosystems to SLR. Therefore, the integration of this impact pathway into the LCIA framework can improve the representation of climate change impacts on biodiversity in particularly affected coastal areas, although the contribution is small at the global scale.^10^

### Implementation and Outlook

Anthropogenic climate change poses a major threat to terrestrial biodiversity, not only through temperature increases but also via indirect effects such as SLR. Therefore, we developed CFs for 244 GHGs with possible differentiation into five taxa and a taxon-generic group. They can be directly integrated into the impact pathway for climate change damages in LCIA by connecting them with GHG emission inventories. As part of this integration, we recommend implementing CFs in Table S7 into existing LCIA methodologies, combining SLR additively with the CF of surface-temperature increase developed by Iordan et al.^23^ We assume SLR impacts are additive to other temperature-induced damages, while acknowledging two limitations: first, combining both may lead to double counting, as surface temperature increases in flooded regions will no longer affect terrestrial species; and second, a simple sum does not capture the complex species responses to both pressures in the remaining habitat. Nevertheless, the combination will still provide a more accurate evaluation of damages from climate change to ecosystem quality at endpoint level.

We further recommend prioritizing global species loss rather than regional species loss and, where possible, using taxon-specific CFs. However, taxon-generic values are also representative, as the differences are comparatively small. Note that species loss is expressed here as the potential fractions of species loss in PDF and not as the actual number of species threatened with extinction.

We developed CFs based on five SSPs, making them climate-scenario-dependent. For prospective LCA studies examining specific future scenarios, the respective CFs can be applied directly. For standard LCA applications and as approximations for practical LCIA implementation, we recommend using the CFs based on SSP2-4.5, which is our default scenario. To support quantitative uncertainty assessment in LCA, we provide mean, minimum, and maximum CFs for each SSP and across SSPs. However, it must be considered that only a limited number of sources of uncertainty could be addressed quantitatively, while others, such as AGTP and SAR parameters, elevation data, and GEP, are assessed qualitatively.

The results of the flood model are not specific to this impact pathway and can also be used to identify general hot spots of future flooding. By using the latest coastal elevation data with corrected sea level height alignment, risks from coastal flooding can be reassessed and targeted conservation and adaptation planning improved. This applies not only to terrestrial biodiversity but also to human health and critical assets.

Despite this major advance in the characterization of climate change damage to terrestrial biodiversity, it is important to note that gaps still remain. For example, SLR models are based on continuous GHG emissions along each SSP, whereas we calculated the AGTP for a single impulse emission. Aligning both is, however, challenging because SLR is a nonlinear process, meaning that a pulse emission at different points in time leads to different SLR outcomes. Although cumulative AGTP values can be calculated for SSPs, allocating them to individual GHGs is difficult. Consequently, our impact pathway combines a pulse-based FF with scenario-dependent XF, which creates a conceptual conflict in its application. For example, the CFs applied to a specific pulse emission will always result in the same impact regardless of its timing and the background warming trajectory, which do not reflect reality. Another gap regarding AGTP is its missing uncertainty assessment. Given the strong influence of the FF on the CFs, we recommend assessing AGTP-related uncertainties in future climate change impact pathways.

Although taxon- and ecoregion-specific parameters and GEP values were applied, important ecological aspects such as the loss of key species, food-web interactions, functional diversity within ecosystems, or ecosystem services are not depicted by the EF and VF. To consider those, different models would be required to calculate species loss or, more generally, damages to ecosystem quality. Furthermore, future adaptation measures to SLR, future land-use conditions, and secondary SLR effects, such as salinization, are excluded here, which may lead to over- or underestimation of SLR-induced damages. In addition, the choice of reference state for the EFs may influence the results and introduce additional uncertainty. Future research could investigate this uncertainty by deriving factors based on alternative reference states, such as natural or projected future conditions. Finally, a linear approach is applied for the development of the impact pathway, which does not always match with nonlinear relationships such as climate change, SLR, coastal flooding, and SAR. Although assumed when applying CFs, the impact of an emission does not necessarily scale proportionally with its magnitude—doubling the emission may not double the impact. Furthermore, when CFs are applied additively, it is implicitly assumed that the marginal impact remains constant across different background states, which may not hold in nonlinear systems. Thus, one starting point for future research is time-resolved CFs. They can better capture the temporal component of this nonlinearity and, in parallel, identify potential periods of abrupt species extinctions.

Overall, SLR damages are not limited to terrestrial biodiversity but are also interlinked with other environmental categories, such as freshwater resources. However, evaluating these interlinkages between environmental categories and impact pathways is rarely done despite being urgently needed. Besides further SLR damage, many additional climate change impact pathways are not yet assessed, not only for terrestrial but also for marine and freshwater species, and we strongly recommend investigating their impacts.

## Supplementary Material





## Data Availability

The Supporting Information is available free of charge as .pdf, .csv, and .tif files in the online version on Zenodo at 10.5281/zenodo.1834059. Code availability: the Python code for the calculations will be made publicly available via the project repository on GitLab (https://gitlab.com/Nele_Teutloff/slr-modeling.git).

## Notes

FF, EF, CF, and results of SSP1-1.9, SSP1-2.6, SSP2-4.5, SSP3-7.0, and SSP5-8.5 (ZIP)
Additional data and processing details for FF and XF; additional results of the flood model per SSP and ecoregion; the flood model validation; results of the spatial analysis; and final EFs and CFs (PDF)

## References

[1] Life Cycle Assessment: theory and Practice, Hauschild, M. Z. ; Rosenbaum, R. K. ; Olsen, S. I. , Eds.; Springer, 2018

[2] Verones F. (2017). LCIA framework and cross-cutting
issues guidance within the UNEP-SETAC
Life Cycle Initiative. J. Clean. Prod..

[3] Hauschild M.Z., Rosenbaum R.K., Olsen S.I. (2024). Summary for policymakers of the thematic
assessment of the interlinkages among biodiversity, water, food and
health (NEXUS assessment) of the Intergovernmental Science-Policy. Platform On Biodiversity And Ecosystem Services.

[4] Cowie R. H., Bouchet P., Fontaine B. (2022). The Sixth Mass Extinction: fact,
fiction or speculation?. Biol. Rev..

[5] Cooley, S. ; Schoeman, D. ; Bopp, L. ; Boyd, P. ; Donner, S. ; Ghebrehiwet, D. ; Ito, S.-I. ; Kiessling, W. ; Martinetto, P. ; Ojea, E. , Climate Change 2022: impacts, Adaptation, and Vulnerability. Contribution of Working Group II to the Sixth Assessment Report of the Intergovernmental Panel on Climate Change; Cambridge University Press: Cambridge, UK and New York, NY, USA, 2022; pp. 379–550.

[6] Parmesan, C. ; Morecroft, M. ; Trisurat, Y. ; Adrian, R. ; Anshari, G. ; Arneth, A. ; Gao, Q. ; Gonzalez, P. ; Harris, R. ; Price, J. , Climate Change 2022: Impacts, Adaptation and Vulnerability. InContribution of Working Group II to the Sixth Assessment Report of the Intergovernmental Panel on Climate Change Cambridge University Press: Cambridge, UK and New York, NY, USA, 2022; pp. 197–377.

[7] Cissé, G. ; McLeman, R. ; Adams, H. ; Aldunce, P. ; Bowen, K. ; Campbell-Lendrum, D. ; Clayton, S. ; Ebi, K. ; Hess, J. ; Huang, C. , Climate Change 2022: impacts, Adaptation and Vulnerability.In Contribution of Working Group II to the Sixth Assessment Report of the Intergovernmental Panel on Climate Change; Cambridge University Press: Cambridge, UK and New York, NY, USA, 2022; pp. 1041–1170.

[8] Huijbregts M. A. J., Steinmann Z. J. N., Elshout P. M. F., Stam G., Verones F., Vieira M., Zijp M., Hollander A., Van Zelm R. (2017). ReCiPe2016: a harmonised life cycle impact assessment
method at midpoint and endpoint level. Int.
J. Life Cycle Assess..

[9] Bulle C., Margni M., Patouillard L., Boulay A.-M., Bourgault G., De Bruille V., Cao V., Hauschild M., Henderson A., Humbert S. (2019). Supporting
Information
section IMPACT World+: a globally regionalized life cycle impact assessment
method. Int. J. Life Cycle Assess..

[10] Verones F. (2020). LC-IMPACT: A regionalized
life cycle damage assessment method. J. Ind.
Ecol..

[11] Li D., Dorber M., Barbarossa V., Verones F. (2022). Global characterization
factors for quantifying the impacts of increasing water temperature
on freshwater fish. Ecol. Indic..

[12] Verones F., Hanafiah M. M., Pfister S., Huijbregts M. A., Pelletier G. J., Koehler A. (2010). Characterization factors for thermal
pollution in freshwater aquatic environments. Environ. Sci. Technol..

[13] Amores M. J., Verones F., Raptis C., Juraske R., Pfister S., Stoessel F., Antón A., Castells F., Hellweg S. (2013). Biodiversity
impacts from salinity increase in a coastal wetland. Environ. Sci. Technol..

[14] Roibás-Rozas A., Núñez M., Mosquera-Corral A., Hospido A. (2022). Modeling the Impact
of Salinity Variations on Aquatic Environments: Including Negative
and Positive Effects in Life Cycle Assessment. Environ. Sci. Technol..

[15] Hanafiah M. M., Leuven R. S., Sommerwerk N., Tockner K., Huijbregts M. A. (2013). Including
the introduction of exotic species in life cycle impact assessment:
The case of inland shipping. Environ. Sci. Technol..

[16] IPCC Climate Change. The Physical Science Basis; IPCC Climate: Cambridge, UK and New York, NY, USA. 2021

[17] Oppenheimer, M. ; Glavovic, B. C. ; Hinkel, J. ; van de Wal, R. ; Magnan, A. K. ; Abd-Elgawad, A. ; Cai, R. ; Cifuentes-Jara, M. ; De Conto, R. M. ; Ghosh, T. , . IPCC Special Report on the Ocean and Cryosphere in a Changing Climate; Cambridge University Press: Cambridge, UK and New York, NY, USA, 2019. pp. 321–445.

[18] Seeger K., Minderhoud P. S. (2026). Sea level much higher than assumed in most coastal
hazard assessments. Nature.

[19] Rosenbaum, R. K. ; Hauschild, M. Z. ; Boulay, A.-M. ; Fantke, P. ; Laurent, A. ; Núñez, M. ; Vieira, M. Life cycle assessment; Springer, 2018; pp. 167–270.

[20] Verones F., Kuipers K. J. J., Núñez M., Rosa F., Scherer L., Marques A., Michelsen O., Barbarossa V., Jaffe B., Pfister S. (2022). Global
extinction probabilities
of terrestrial, freshwater, and marine species groups for use in Life
Cycle Assessment. Ecol. Indic..

[21] IPCC Climate Change The Physical Science Basis; IPCC Climate Change: Cambridge, UK and New York, NY, USA, 2013.

[22] Hanafiah M. M., Xenopoulos M. A., Pfister S., Leuven R. S., Huijbregts M. A. (2011). Characterization
factors for water consumption and greenhouse gas emissions based on
freshwater fish species extinction. Environ.
Sci. Technol..

[23] Iordan C.-M., Kuipers K. J. J., Huang B., Hu X., Verones F., Cherubini F. (2023). Spatially and taxonomically explicit
characterisation
factors for greenhouse gas emission impacts on biodiversity. Resour. Conserv. Recycl..

[24] Jolliet O., Antón A., Boulay A. M., Cherubini F., Fantke P., Levasseur A., McKone T. E., Michelsen O., Milàicanals L., Motoshita M., Pfister S., Verones F., Vigon B., Frischknecht R. (2018). Global guidance on environmental
life cycle impact assessment indicators: impacts of climate change,
fine particulate matter formation, water consumption and land use. Int. J. Life Cycle Assess..

[25] Levasseur, A. ; de Schryver, A. ; Hauschild, M. ; Kabe, Y. ; Sahnoune, A. ; Tanaka, K. ; Cherubini, F. Global Guidance for Life Cycle Impact Assessment Indicators; UNEP/SETAC Life Cycle Initiative, 2016; pp. 58–75.

[26] IPCC Climate Change. The Physical Science Basis; IPCC, 2021.

[27] Zieger, V. ; Lecompte, T. ; Guihéneuf, S. ; Guevel, Y. ; Bazzana, M. ; Gasser, T. ; He, Y. Climate change metrics: IPCC AR6 updates, discussions and dynamic assessment applications EGUsphere 2025

[28] IPCC Climate Change. The Physical Science Basis; IPCC Climate Change, 2013pp. 1–44.

[29] Hermans T. H., Gregory J. M., Palmer M. D., Ringer M. A., Katsman C. A., Slangen A. B. (2021). Projecting global mean sea-level change using CMIP6
models. Geophys. Res. Lett.

[30] Kopp R. E., Garner G. G., Hermans T. H., Jha S., Kumar P., Reedy A., Slangen A. B., Turilli M., Edwards T. L., Gregory J. M. (2023). The Framework for Assessing Changes To Sea-level
(FACTS) v1. 0: a platform for characterizing parametric and structural
uncertainty in future global, relative, and extreme sea-level change. EGU European Geosciences Union.

[31] Garner, G. G. , IPCC AR6 Sea Level Projections. Version 20210809.

[32] IPCC, IPCC WGI Interactive Atlas. https://interactive-atlas.ipcc.ch/, Accessed: 2022–09–01.

[33] IPCC,CEDA Archive - Summary for Policymakers of the Working Group I Contribution to the IPCC Sixth Assessment Report - data for Figure SPM.8. Accessed 11.09.2025; https://data.ceda.ac.uk/badc/ar6_wg1/data/spm/spm_08/v20210809/panel_a.

[34] Yunus A. P., Avtar R., Kraines S., Yamamuro M., Lindberg F., Grimmond C. S. B. (2016). Uncertainties
in tidally adjusted estimates of sea
level rise flooding (bathtub model) for the greater London. Remote Sens..

[35] Kasmalkar I., Wagenaar D., Bill-Weilandt A., Choong J., Manimaran S., Lim T. N., Rabonza M., Lallemant D. (2024). Flow-tub model:
A modified bathtub flood model with hydraulic connectivity and path-based
attenuation. MethodsX.

[36] Pronk M., Hooijer A., Eilander D., Haag A., de Jong T., Vousdoukas M., Vernimmen R., Ledoux H., Eleveld M. (2024). DeltaDTM:
A global coastal digital terrain model. Sci.
Data.

[37] Scholz R. (2007). Assessment
of Land Use Impacts on the Natural Environment. Part 1: An Analytical
Framework for Pure Land Occupation and Land Use Change (8. Int. J. Life Cycle Assess.

[38] Olson D. M. (2001). Terrestrial Ecoregions of the World: A New
Map of Life on EarthA
new global map of terrestrial ecoregions provides an innovative tool
for conserving biodiversity. BioScience.

[39] Pfister S., Oberschelp C., Sonderegger T. (2020). Regionalized LCA in practice: the
need for a universal shapefile to match LCI and LCIA. Int. J. Life Cycle Assess.

[40] WWF. World Wildlife Fund Terrestrial Ecoregions of the World. https://www.worldwildlife.org/publications/terrestrial-ecoregions-of-the-world, Accessed 2025–09–07.

[41] Arrhenius O. (1921). Species and
area. J. Ecol..

[42] Rosenzweig, M. L. Species Diversity in Space and Time; Cambridge University Press: Cambridge, UK, 1995.

[43] Chaudhary A., Verones F., De Baan L., Hellweg S. (2015). Quantifying Land Use
Impacts on Biodiversity: Combining Species-Area Models and Vulnerability
Indicators. Environ. Sci. Technol..

[44] Dorber M., Kuipers K., Verones F. (2020). Global characterization
factors for
terrestrial biodiversity impacts of future land inundation in Life
Cycle Assessment. Sci. Total Environ..

[45] Scherer L., Rosa F., Sun Z., Michelsen O., De Laurentiis V., Marques A., Pfister S., Verones F., Kuipers K. J. J. (2023). Biodiversity impact assessment considering
land use
intensities and fragmentation. Environ. Sci.
Technol..

[46] Kuipers K. J., Hellweg S., Verones F. (2019). Potential consequences of regional
species loss for global species richness: a quantitative approach
for estimating global extinction probabilities. Environ. Sci. Technol..

[47] Verones, F. ; Kuipers, K. J. J. ; Núñez, M. ; Rosa, F. ; Scherer, L. ; Marques, A. ; Michelsen, O. ; Barbarossa, V. ; Jaffe, B. ; Pfister, S. , Global extinction probabilities of terrestrial, freshwater, and marine species groups. https://zenodo.org/records/14499582, 2024.

[48] Hausfather Z., Peters G. P. (2020). Emissions–the
‘business as usual’
story is misleading. Nature.

[49] Van
Alphen J. (2016). The Delta Programme and updated flood risk management policies in
the Netherlands. J. Flood Risk Manag..

[50] Collini, R. C. ; Carter, J. ; Auermuller, L. ; Engeman, L. ; Hintzen, K. ; Gambill, J. ; Johnson, R. E. ; Miller, I. ; Schafer, C. ; Stiller, H. . Application Guide for the 2022 Sea Level Rise Technical Report; National Oceanic and Atmospheric Administration (NOAA) Office for Coastal Management, 2022.

[51] Southeast Florida Regional Climate Change Compact Sea Level Rise Work Group, Unified Sea Level Rise Projection: southeast Florida, 2019 Update; Southeast Florida Regional Climate Change Compact, 2020.

[52] Infrastructure Australia, Coastal Hazards Adaptation Strategy; 2019.

[53] Magnan A. K. (2023). Status of global coastal adaptation. Nat. Clim.
Chang..

[54] Earth, O. Bahamian-Antillean Mangroves. https://www.oneearth.org/ecoregions/bahamian-antillean-mangroves/, Accessed: 26.03.2026.

[55] Kier G., Kreft H., Lee T. M., Jetz W., Ibisch P. L., Nowicki C., Mutke J., Barthlott W. (2009). A global assessment
of endemism and species richness across island and mainland regions. Proc. Natl. Acad. Sci. U. S. A..

[56] United Nations Kyoto protocol to the united nations framework convention on climate change; United Nations, 1997

[57] Levasseur A., Cavalett O., Fuglestvedt J. S., Gasser T., Johansson D. J. A., Jørgensen S. V., Raugei M., Reisinger A., Schivley G., Strømman A., Tanaka K., Cherubini F. (2016). Enhancing
life cycle impact assessment from climate science: Review of recent
findings and recommendations for application to LCA. Ecol. Indic..

