# Supplementary material for: Sea Level Rise Damages on Ecosystem Quality—A Life Cycle Impact Pathway
Source: Environ Sci Technol. 2026 Sep 28;60(40):28566–76. doi: 10.1021/acs.est.6c04798 (PMC13674083; doi:10.1021/acs.est.6c04798)
Supplement: Supplementary file 1 [file es6c04798_si_002.pdf]

Supporting Information: Sea level rise damages on ecosystem quality – a life cycle impact pathway

Nele Teutloff,[*,†,‡] Martin Dorber,[¶] Peter K.R. Holzapfel,[†] Matthias Finkbeiner,[†] and Alexis Laurent[‡]

†*Chair of Sustainable Engineering, Institute of Environmental Technology, Technische Universität Berlin, Berlin, 10623, Germany*

‡*Section for Quantitative Sustainability Assessment, Department of Environmental and Resource Engineering, Technical University of Denmark, Lyngby, 2800, Denmark*

¶*Department of Energy and Process Engineering, Norwegian University of Science and Technology, Trondheim, Trøndelag, NO-7491, Norway*

E-mail: n.teutloff@tu-berlin.de

Contents

Fate Factor Calculation 4

Exposure Factor 5

XF1: Cumulative temperature increase per SSP 5

XF2: Flood model results 5

XF2: Flood model validation 8

Effect Factor: Spatial Analysis 11

Combined Characterization Factors 17

List of Figures

S.1 Flooded areas by SLR or extreme sea level for Zealand in 2100. In blue the self-modelled flooding, in green the flooding modelled by Kirezci et al. (2020), in pink the model developed by Scalgo. A: Comparison between the own model and Scalgo B: Comparison between the own model and Kirezci et al., both for entire Zealand. C & D: Zoom into a representative area marked in A & B as a circle. 11

S.2 Regional species loss (SLR·EF) by 2100 under SSP2 for amphibians [PDF] 12

S.3 Regional species loss (SLR·EF) by 2100 under SSP2 for reptiles [PDF] 13

S.4 Regional species loss (SLR·EF) by 2100 under SSP2 for vascular plants [PDF] . . 13

S.5 Regional species loss (SLR·EF) by 2100 under SSP2 for mammals [PDF] 14

S.6 Regional species loss (SLR·EF) by 2100 under SSP2 for birds [PDF] 14

S.7 Global species loss (SLR·EF·VF) by 2100 under SSP2 for amphibians [PDF] . . . 15

S.8 Global species loss (SLR·EF·VF) by 2100 under SSP2 for reptiles [PDF] 15

S.9 Global species loss (SLR·EF·VF) by 2100 under SSP2 for vascular plants [PDF] . 16

S.10 Global species loss (SLR·EF·VF) by 2100 under SSP2 for mammals [PDF] 16

S.11 Global species loss (SLR·EF·VF) by 2100 under SSP2 for birds [PDF] 17

List of Tables

S1 Coefficients for AGTP calculation based on Zieger et al. (2025)[1] 4
S2 Additional coefficients for AGTP calculation for CO2[1] 5
S3 Cumulative temperature increase by 2100 [K·yrs] for five SSPs 5
S4 Summary of flood model results for three SSP 7
S5 Summary of flood model results for two SSP 8
S6 Main properties of flood models and their projected flooded area on zealand by 2100[2–4] 9
S7 Combined CFs for surface temperature increase[5] and SLR [PDF·yr/kg] . . . 18

Fate Factor Calculation

Equation S1: AGTP for non-CO2 GHGs from IPCC's Fifths Assessment Report (AR5) supporting information[6]

$$AGTP_i(H) = A_i \sum_{j=1}^{2} \frac{\tau c_j}{\tau - d_j} \left(e^{-H/\tau} - e^{-H/d_j} \right) \tag{S1}$$

Equation S2: Time-integrated AGTP for non-CO2 GHGs based on Equation S1

$$iAGTP_i(H) = A_i \sum_{j=1}^{2} \frac{\tau c_j}{\tau - d_j} \left(-\tau e^{-H/\tau} + \tau + d_j e^{-H/d_j} - d_j \right) \tag{S2}$$

where $AGTP_i(H)$ is the AGTP for a non-CO2 GHG i with a time horizon of H, A_i represents the radiative efficiency of the gas, τ_i is the GHG lifetime, and c_j and d_j are coefficients of the temperature response function.

Table S1: Coefficients for AGTP calculation based on Zieger et al. (2025)[1]

**Coefficient**	**Value**	**Unit**
c1	0.586	K/Wm^2
c2	0.414	K/Wm^2
d1	3.4	Years
d2	285	Years

Equation S3: AGTP for CO2 from IPCC's Fifths Assessment Report (AR5) supporting information[6]

$$AGTP_{CO2}(H) = A_{CO2} \sum_{j=1}^{2} \left(\alpha_0 c_j \left(1 - e^{-H/d_j} \right) + \sum_{i=3}^{3} \frac{a_i \tau_i c_j}{\tau_i - d_j} \left(e^{-H/\tau_i} - e^{-H/d_j} \right) \right) \tag{S3}$$

Equation S4: Time-integrated AGTP for CO2 based on IPCC's Fifths Assessment Report (AR5) supporting information[6]

$$\text{iAGTP}_{\text{CO}_2}(H) = A_{\text{CO}_2} \sum_{j=1}^{2} \left[\alpha_0 c_j \left(H + d_j e^{-H/d_j} - d_j \right) + \sum_{i=1}^{3} \frac{\alpha_i \tau_i c_j}{\tau_i - d_j} \left(-\tau_i e^{-H/\tau_i} + \tau_i + d_j e^{-H/d_j} - d_j \right) \right] \tag{S4}$$

Where AGTP$_{CO2}$(H) is the AGTP for a CO2 with a time horizon of H, A$_{CO2}$ represents the radiative efficiency of CO2, τ_i are the time scales, and a$_i$, c$_j$, and d$_j$ are coefficients of the temperature response function.

Table S2: Additional coefficients for AGTP calculation for CO2[1]

**Coefficient**	**i=0**	**i=1**	**i=2**	**i=3**	**Unit**
α_i	0.2173	0.2240	0.2824	0.2763	Unitless
τ_i		394.4	36.54	4.304	Years

Exposure Factor

XF1: Cumulative temperature increase per SSP

The CMIP6 multimodel is used by IPCC to calculate the surface temperature increase per Shared Socioeconomic Pathways (SSP) until 2100. It gives the mean, 5% and 95% percentile for the surface temperature increase for each year until 2100. The calculated cumulative temperature increase for each SSP and percentile is given in Table S3. The data used for the calculation can be found here: `https://data.ceda.ac.uk/badc/ar6_wg1/data/spm/spm_08/v20210809/panel_a`

Table S3: Cumulative temperature increase by 2100 [K·yrs] for five SSPs

	5% [K·yrs]	Mean [K·yrs]	95% [K·yrs]
SSP1 1.9	99.9	123.8	151.4
SSP1 2.6	112.8	139.9	175.2
SSP2 4.5	141.8	173.3	214.8
SSP3 7.0	166.0	203.7	251.2
SSP5 8.5	185.2	234.3	293.3

XF2: Flood model results

The relative sea level rise (SLR) by 2100 is modeled with the CMIP6 multimodel. It is the relative SLR by 2100 compared to the reference sea level from 1995-2014. The model is based on the surface temperature increase from the SSPs (globally averaged surface air temperature over the period 2081-2100 compared to temperature from 1850-1900). The SLR-model has as an output the SLR in m and a spatially resolution of 1°. (`https://interactive-atlas.ipcc.ch/`). It is used as an input for the developed flood model together with the corrected DeltaDTM.[7] The DeltaDTM derives the terrain elevations in mm scale at a 1-seconds resolution (approximately 30 m at the equator) and covers land areas between the latitudes of 90N-60S. The results of the flood model are summarized in Table S4 and Table S5.

Table S4: Summary of flood model results for three SSP

	**SSP1 1.9**	**SSP1 2.6**	**SSP2 4.5**
affected ecoregions count	459	459	459
ecoregion with min flood (km2)	Corsican montane broadleaf and mixed forests	Corsican montane broadleaf and mixed forests	Eastern Zimbabwe montane forest-grassland mosaic
min flooded area (km2)	1.8E-3	1.8E-3	2.3E-3
ecoregion with min flood (%)	East Siberian taiga	East Siberian taiga	East Siberian taiga
min flooded area [%]	4.6E-7	5.2E-7	1.0E-6
ecoregion with median flood (km2)	San Lucan xeric scrub	Lara-Falcón dry forests	Antipodes Subantarctic Islands tundra
median flooded area (km2)	78.6	87.5	103.4
ecoregion with median flood (%)	Hawaii tropical low shrublands	Deccan thorn scrub forests	Eastern Java-Bali montane rain forests
median flooded area (%)	0.18	0.19	0.25
ecoregion with max flood (km2)	Western Gulf coastal grasslands	Western Gulf coastal grasslands	Atlantic mixed forests
max flooded area (km2)	16646.3	16854.7	17387.2
ecoregion with max flood (%)	Mesoamerican Gulf-Caribbean mangroves	Mesoamerican Gulf-Caribbean mangroves	Central African mangroves
max flooded area (%)	34.1	35.6	40.8
total flooded area (km2)	249,301	270,717	319,359

Table S5: Summary of flood model results for two SSP

	**SSP3 7.0**	**SSP5 8.5**
affected ecoregions count	460	460
ecoregion with min flood [km2]	Eastern Zimbabwe montane forest-grassland mosaic	Eastern Zimbabwe montane forest-grassland mosaic
min flooded area [km2]	2.3E-3	2.3E-3
ecoregion with min flood [%]	East Siberian taiga	East Siberian taiga
min flooded area [%]	1.7E-6	2.6
ecoregion with median flood [km2]	Andaman Islands rain forests	Sulu Archipelago rain forests
median flooded area [km2]	123.2	142.2
ecoregion with median flood [%]	Westland temperate forests	Faroe Islands boreal grasslands
median flooded area [%]	0.28	0.31
ecoregion with max flood [km2]	Atlantic mixed forests	Atlantic mixed forests
max flooded area [km2]	18228.2	19388.7
ecoregion with max flood [%]	Indochina mangroves	Indochina mangroves
max flooded area [%]	46.8	52.5
total flooded area [km2]	371,704	420,638

XF2: Flood model validation

To validate the flooding model, we compared the results compared to two other SLR flood models. However, both models differ in either their extent, data selection, or approach. The first model is developed by the Danish company Scalgo and is only available in Nordic countries: Denmark, Poland, Germany, and the UK.[8] The second model, by Kirezci et al. (2020), covers global flooding but for extreme sea levels.[4] The results were compared for a representative area in Denmark: Zealand. We selected this specific area due download restriction of Scalgo's model to a size of 500 km^2 and its limited accessibility to Denmark through a DTU student account.

Scalgo: Scalgo is a company that provides tools for high-resolution modeling of surface water and flooding. One of their tools is a SLR analysis calculating the areas potentially

flooded at a given water level. The water level can be adjusted manually and hydrological connectivity is included.[9] For the comparison, we set the sea level to the sea level predicted around Zealand under SSP5 in 2100 (0.58 m).[10] Scalgo uses the high-resolution Danish elevation model (DHM/DK-DEM) with a 0.4 m resolution and 0.05 m vertical precision.[2,11] The data for Zealand, including surrounding island, was downloaded in 14 pieces due to the download restriction. We merged and reclassified the pieces to fit to the self-modeled data in their values for flooded areas using QGIS.

Extreme Sea Levels by Kirezci et al. (2020): Kirezci et al. (2020) developed a model that projects the flooding due to extreme sea levels in 2050 and 2100. They used data from two of the IPCC's Representative Concentration Pathways (RCPs) 4.5 and 8.5 and included tide models, storm surges, and wave setup in their model. Thus, it is not a permanent flooding but episodical. Like the model developed in this study, Kirezci et al. used a bathtub approach with hydrological connectivity and the Multi-Error-Removed Improved-Terrain DEM (MERIT DEM) in a resolution of 1 km at the Equator.[3,4]

Table S6: Main properties of flood models and their projected flooded area on zealand by 2100[2–4]

Model	Resolution	Vertical Precision	Sea Level in 2100	Elevation Model	Flooded Area [km^2]
Scalgo	0.4 m	0.05 m	0.58	DHM	31.18
Kirezci et al.	1 km	58% <2 m	1.5-2.5 m	MERIT DEM	48,271.06
Self-modeled	30 m	0.45 m	0.58 m	DeltaDTM	179.24

Table S6 shows relevant model properties and compares the areas flooded on Zealand calculated with the three models. The models show significant differences in flooded areas, with Scalgo predicting smaller areas along the coastline while the model from Kriezci et al. is showing larger inland areas. The model developed in this study generally shows more overlapping areas with Scalgo's model than with Kriezci et al.. The differences and similarities in flooded area are most likely attributed to three main factors:

1. Higher resolution models can better differentiate elevation and thereby flooding. In models with lower resolution, grid cells cover larger areas which are projected to be flooded if the

elevation is below sea level. Within the three examples it can be seen, that with decreasing resolution the flooded area is increasing.

2. Different elevation models can lead to differences in flood modeling, particularly for sea levels ranging in the magnitude of centimeters. Scalgo uses a specialized elevation model for Denmark with a higher vertical precision than the other two models. Furthermore, the elevation is directly measured by laser scanning of Denmark from airplanes[11] while MERIT DEM and DeltaDTM are spaceborne geophysical global elevation models partly supplemented with spaceborne lidar.[12,13] These models are not automatically aligned with actual local sea levels and need a vertical datum conversion to be used for SLR modeling.[7] In this study, we therefore use the converted DeltaDTM aligning coastal elevation to the latest coastal sea-level height for a 500 km-wide coastal zone.[7] Although elevation models of higher resolution, such as from Scalgo, may achieve more accurate results for regional modeling, they are impractical for large scale modeling due to high computational demands.

3. The assumed sea level leads to significant differences. While Scalgo's sea level could be adjusted to the the sea level of this study, Kirezci et al. uses extreme sea levels.[4]

All three models consider hydrological connectivity but exclude future coastal defenses. Despite the differences, all three models show similar areas at risk, but with variations in the extent of flooding. In Figure S.1 (C) and (D), the differences and similarities between the three models are shown on the example of a smaller area of Zealand. The areas in green, blue, and pink show the flooding by Kirezci et al., the self-developed model, and Scalgo's model, respectively. White areas represent either the sea or areas below sea level but not connected to the sea (inland areas). The selected area shown in detail is marked with a circle in the overview figures of Zealand, see Figure S.1 (A & B).

Overall, the results confirm the robustness of the developed flood model, as the differences from other models are expected and reasonable. The model by Kirezci et al. shows higher flooding due to higher sea levels and lower resolution while the Scalgo's model show lower flooding due its higher resolution and more accurate DEM. Our model lies in between with

a higher similarity to Scalgo's model. Differences in results due to DEM resolutions and DEM selection is a known phenomena and was previously discussed by, for example, Li et al. (2010).[14] Besides these differences, the models show still similar results in the areas flooded with different flooding extents which confirms the effectiveness and robustness of the developed modeling approach.

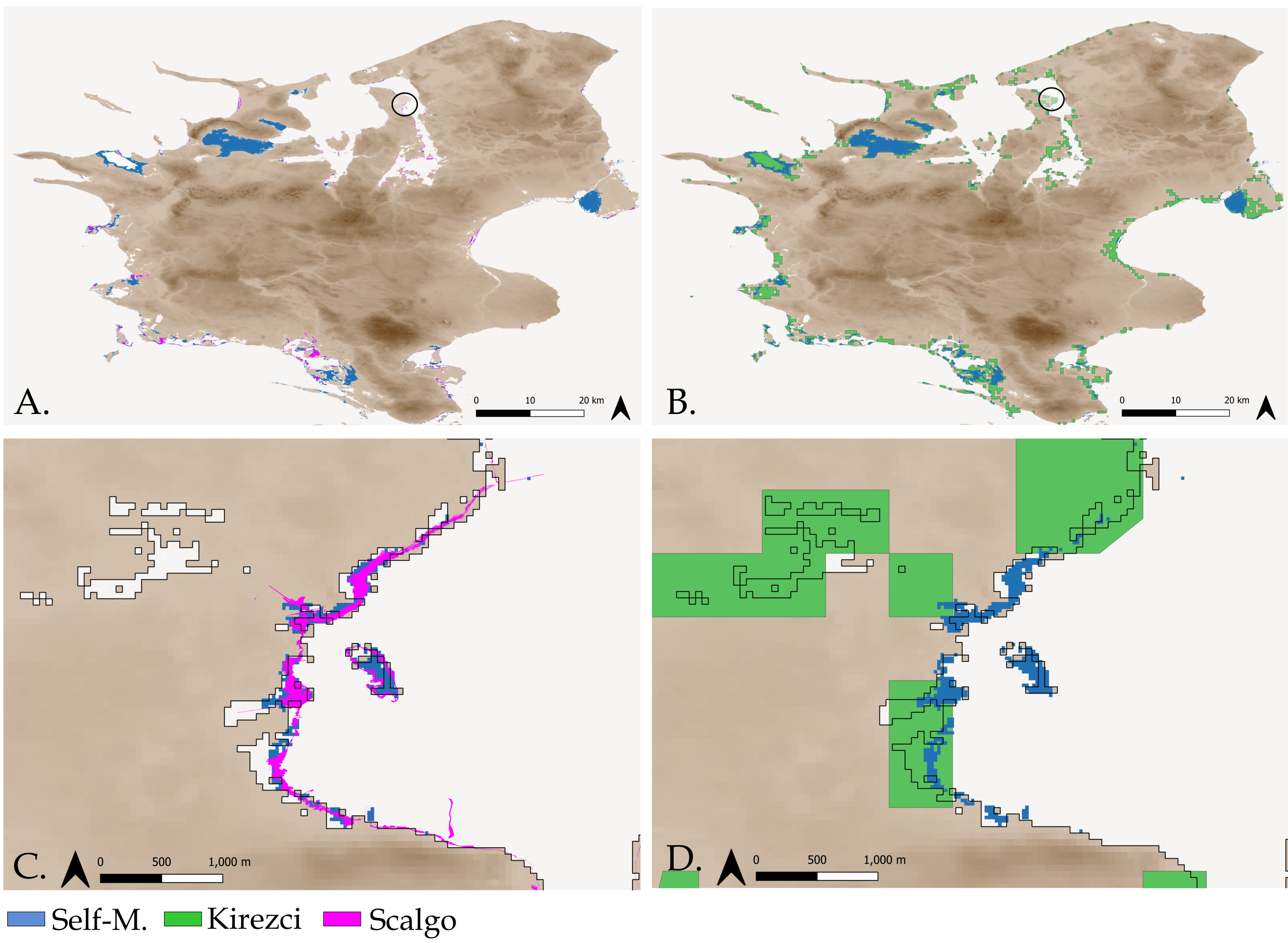

Figure S.1: Flooded areas by SLR or extreme sea level for Zealand in 2100. In blue the self-modelled flooding, in green the flooding modelled by Kirezci et al. (2020), in pink the model developed by Scalgo. A: Comparison between the own model and Scalgo B: Comparison between the own model and Kirezci et al., both for entire Zealand. C & D: Zoom into a representative area marked in A & B as a circle.

Effect Factor: Spatial Analysis

The results of the spatial analysis are depicted here for each taxon and for taxon-specific values. The results are based on the flood model outcome for SSP2 and the EF multiplied with the specific flooded area. Thus, the values here are in PDF and show the potential species loss by 2100 under SSP2.

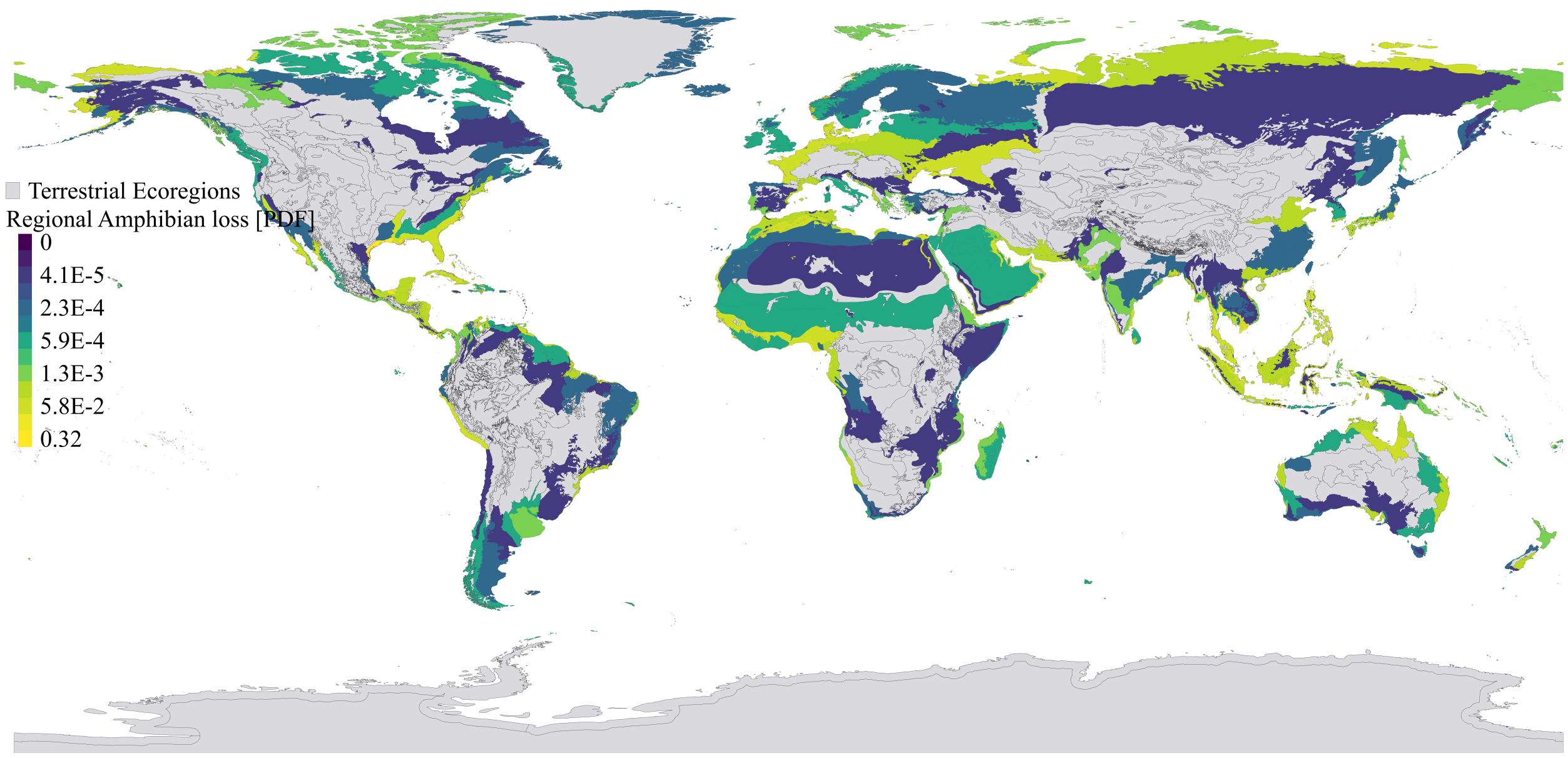

Figure S.2: Regional species loss (SLR·EF) by 2100 under SSP2 for amphibians [PDF]

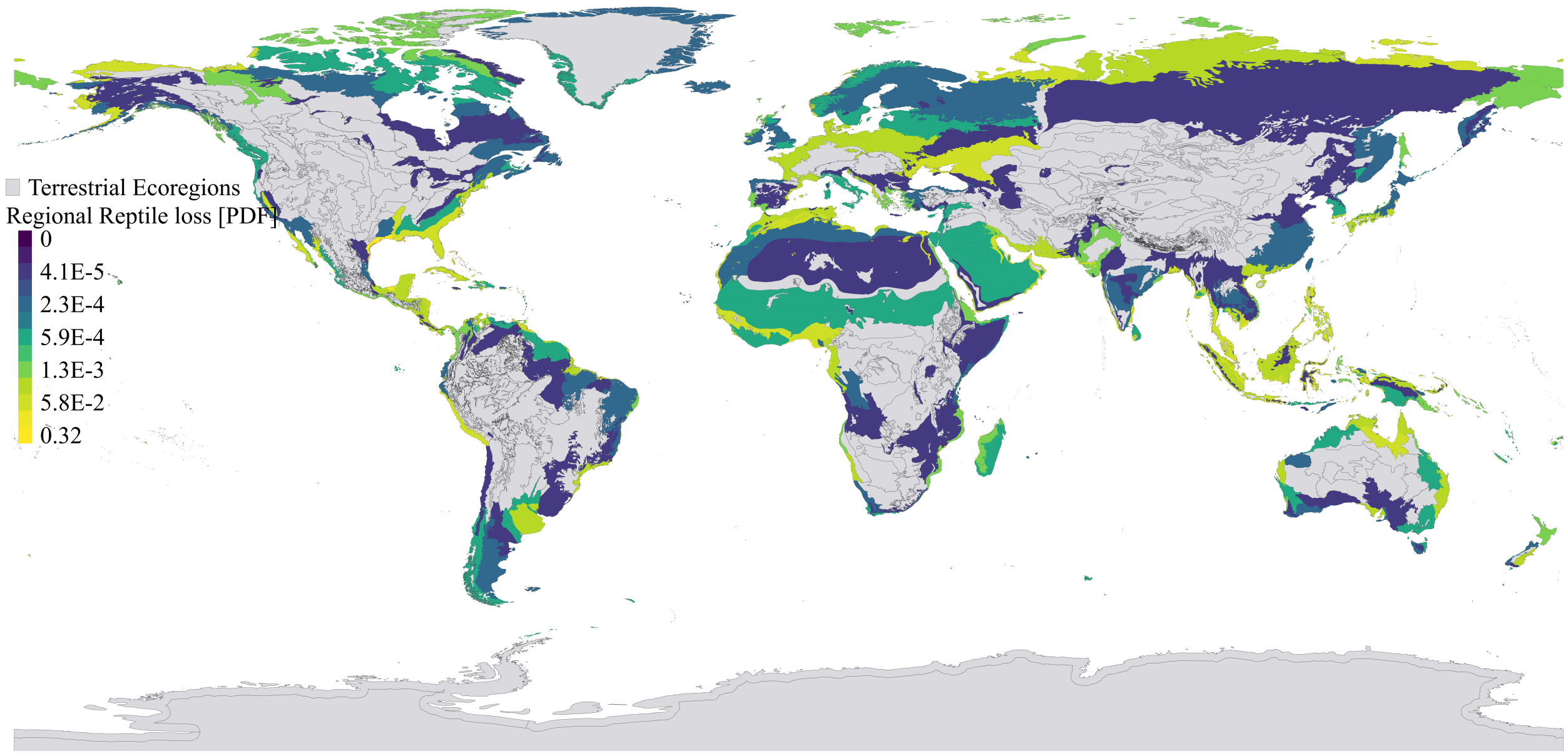

Figure S.3: Regional species loss (SLR·EF) by 2100 under SSP2 for reptiles [PDF]

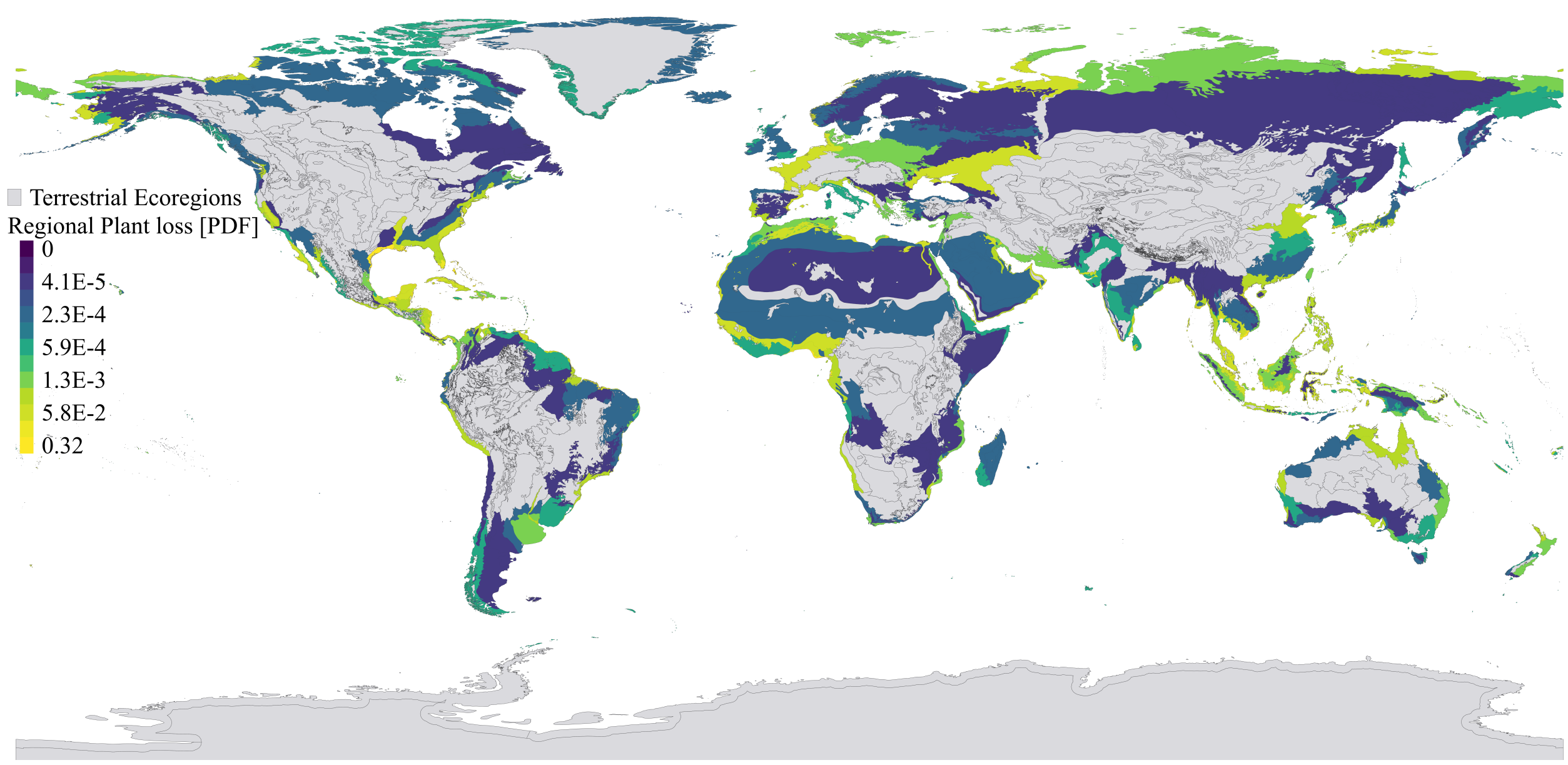

Figure S.4: Regional species loss (SLR·EF) by 2100 under SSP2 for vascular plants [PDF]

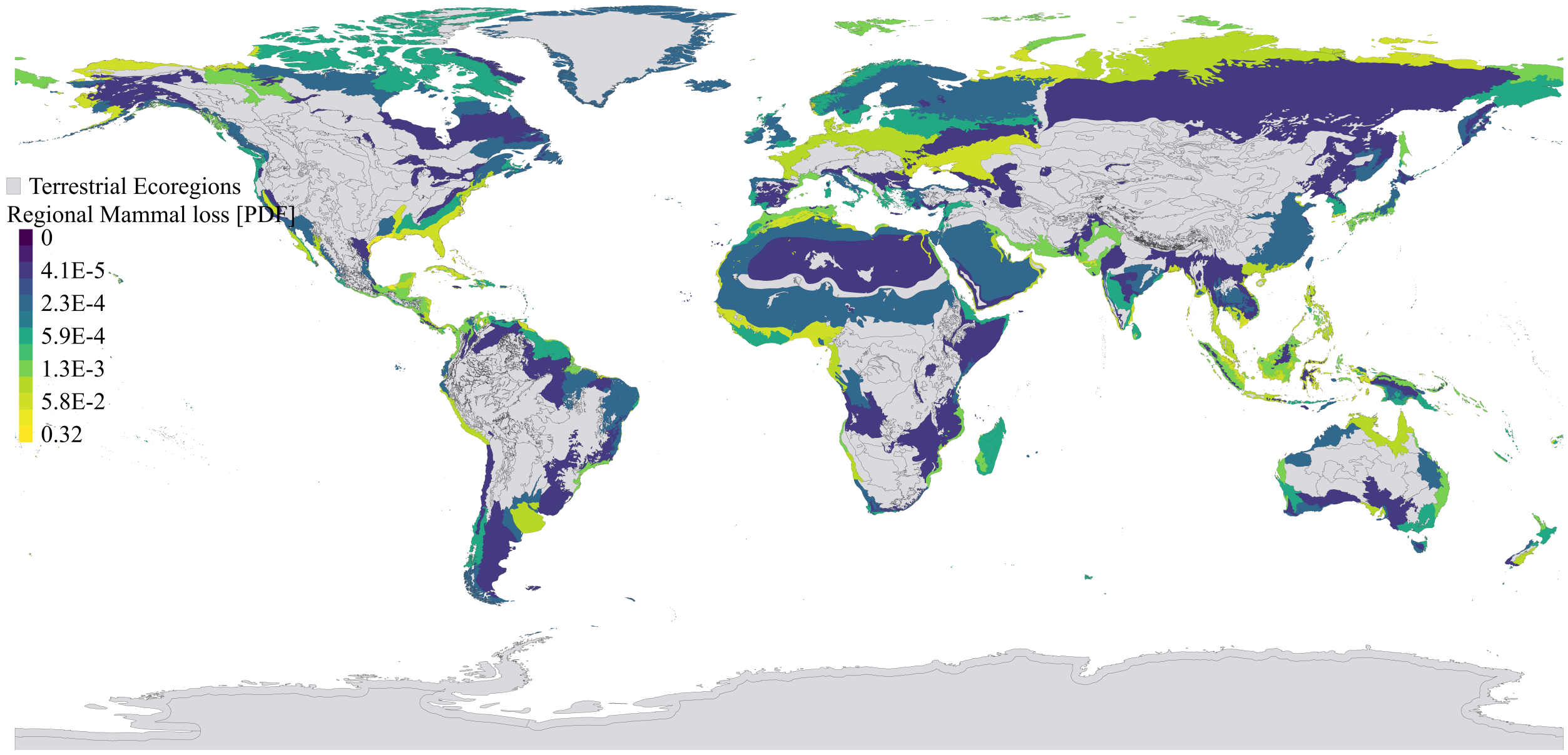

Figure S.5: Regional species loss (SLR·EF) by 2100 under SSP2 for mammals [PDF]

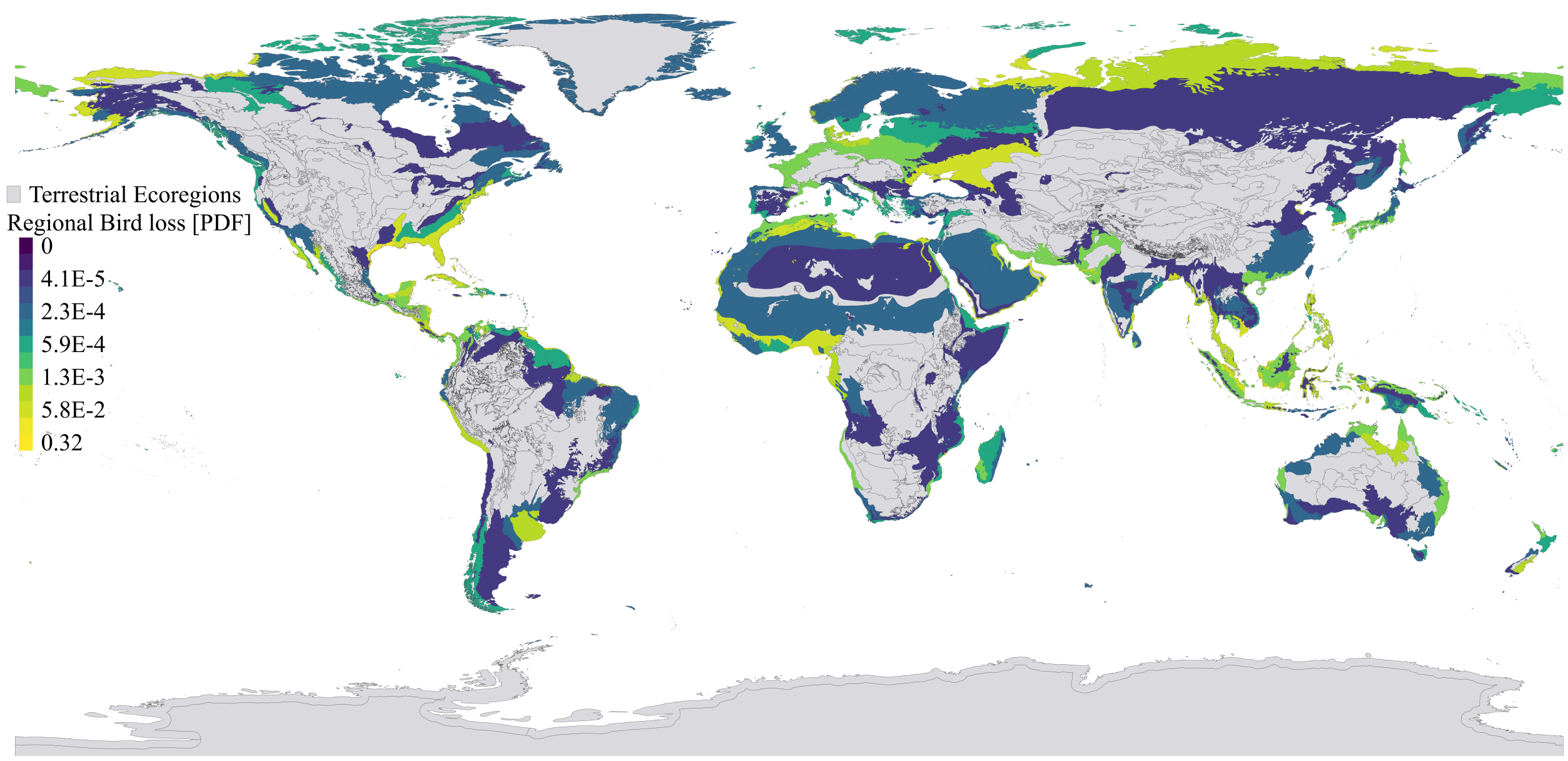

Figure S.6: Regional species loss (SLR·EF) by 2100 under SSP2 for birds [PDF]

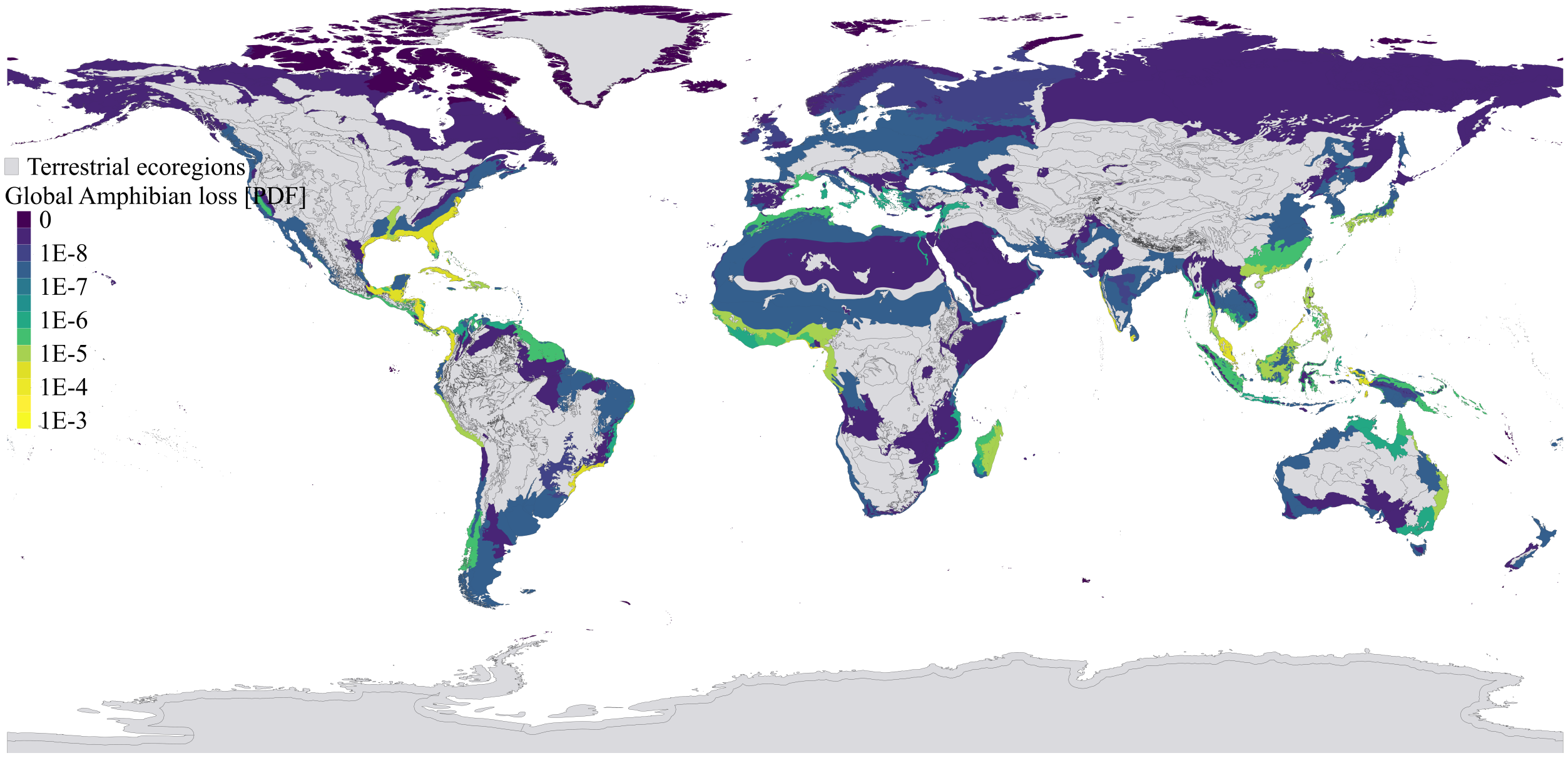

Figure S.7: Global species loss (SLR·EF·VF) by 2100 under SSP2 for amphibians [PDF]

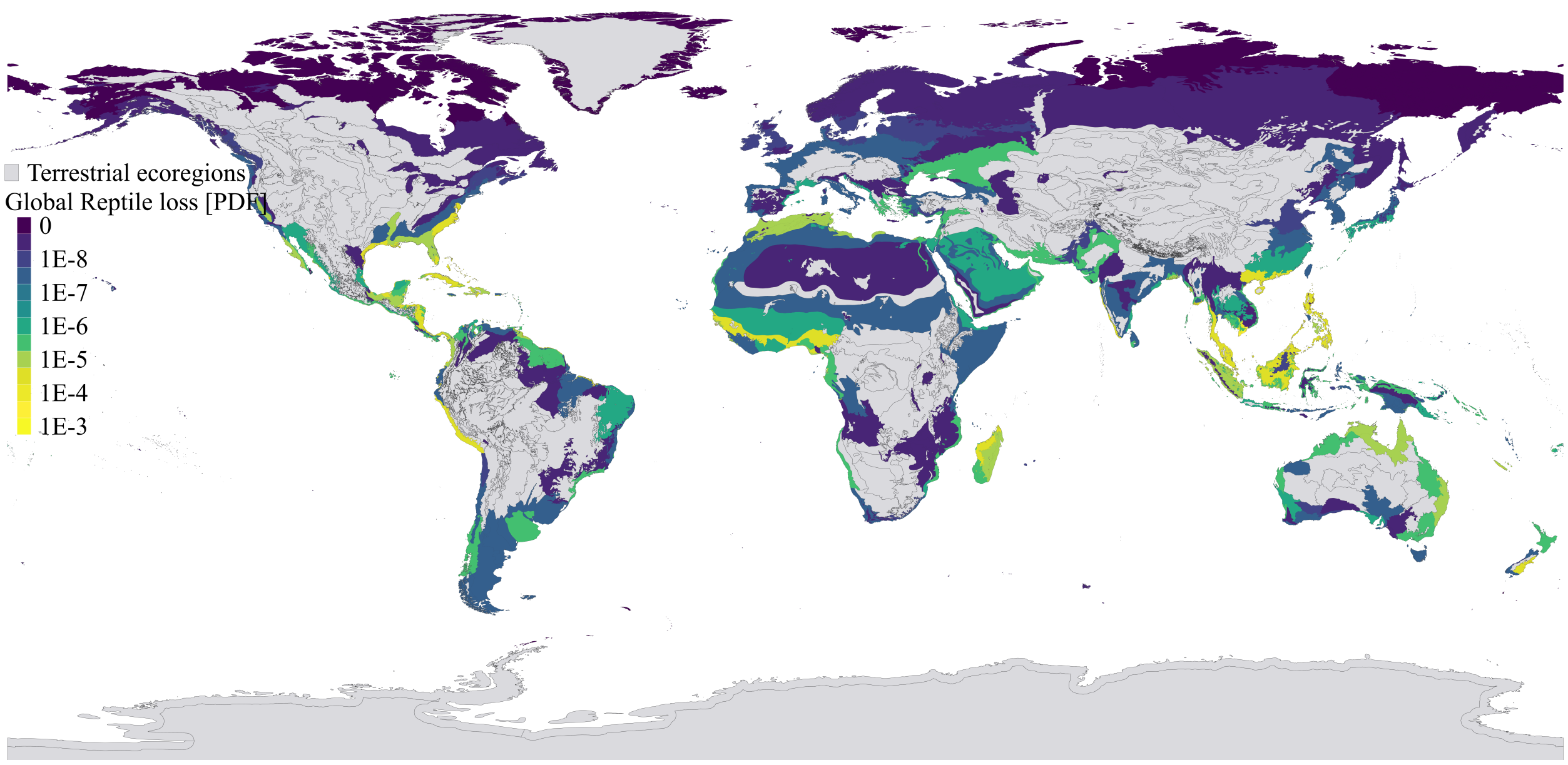

Figure S.8: Global species loss (SLR·EF·VF) by 2100 under SSP2 for reptiles [PDF]

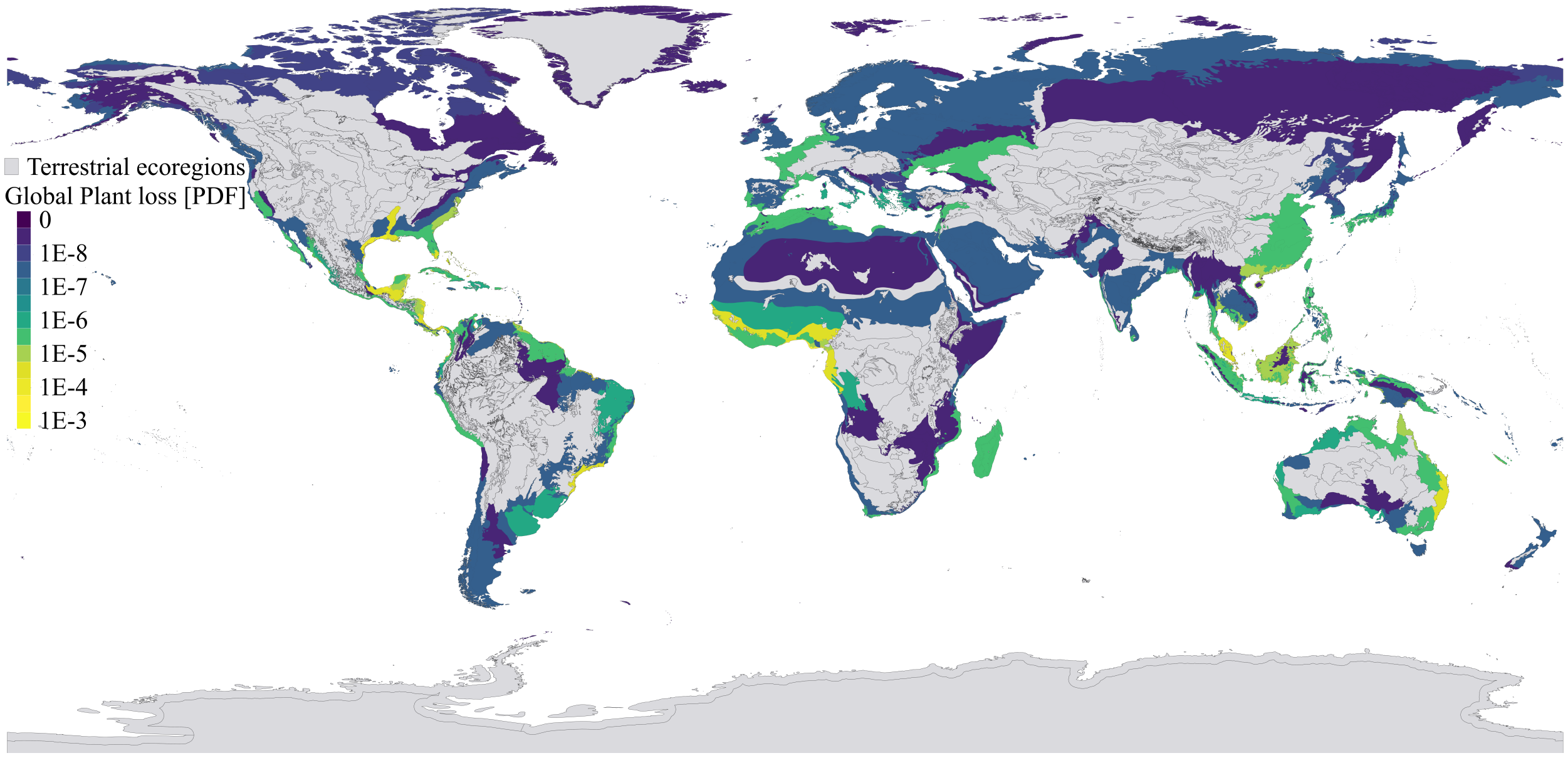

Figure S.9: Global species loss (SLR·EF·VF) by 2100 under SSP2 for vascular plants [PDF]

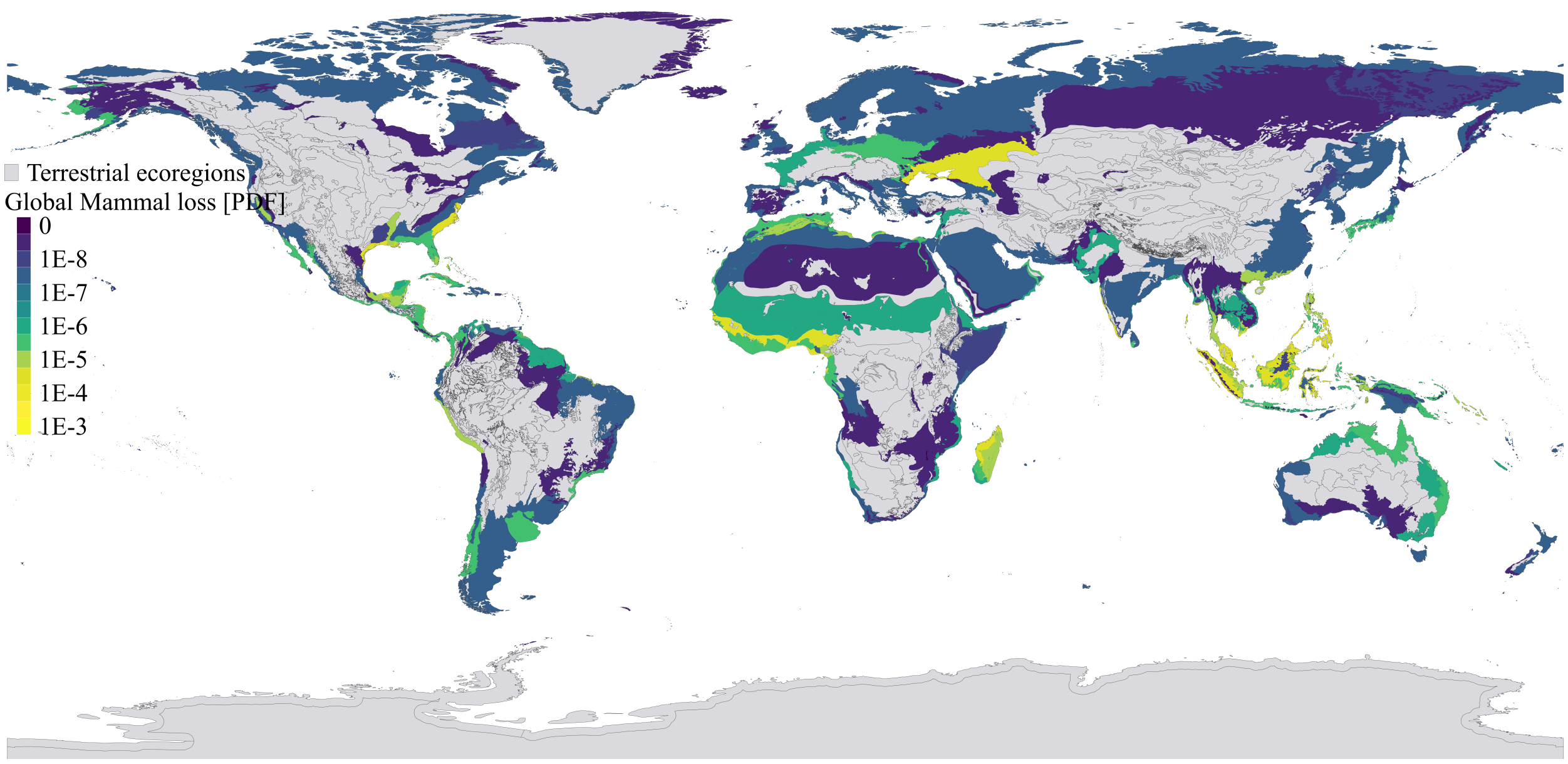

Figure S.10: Global species loss (SLR·EF·VF) by 2100 under SSP2 for mammals [PDF]

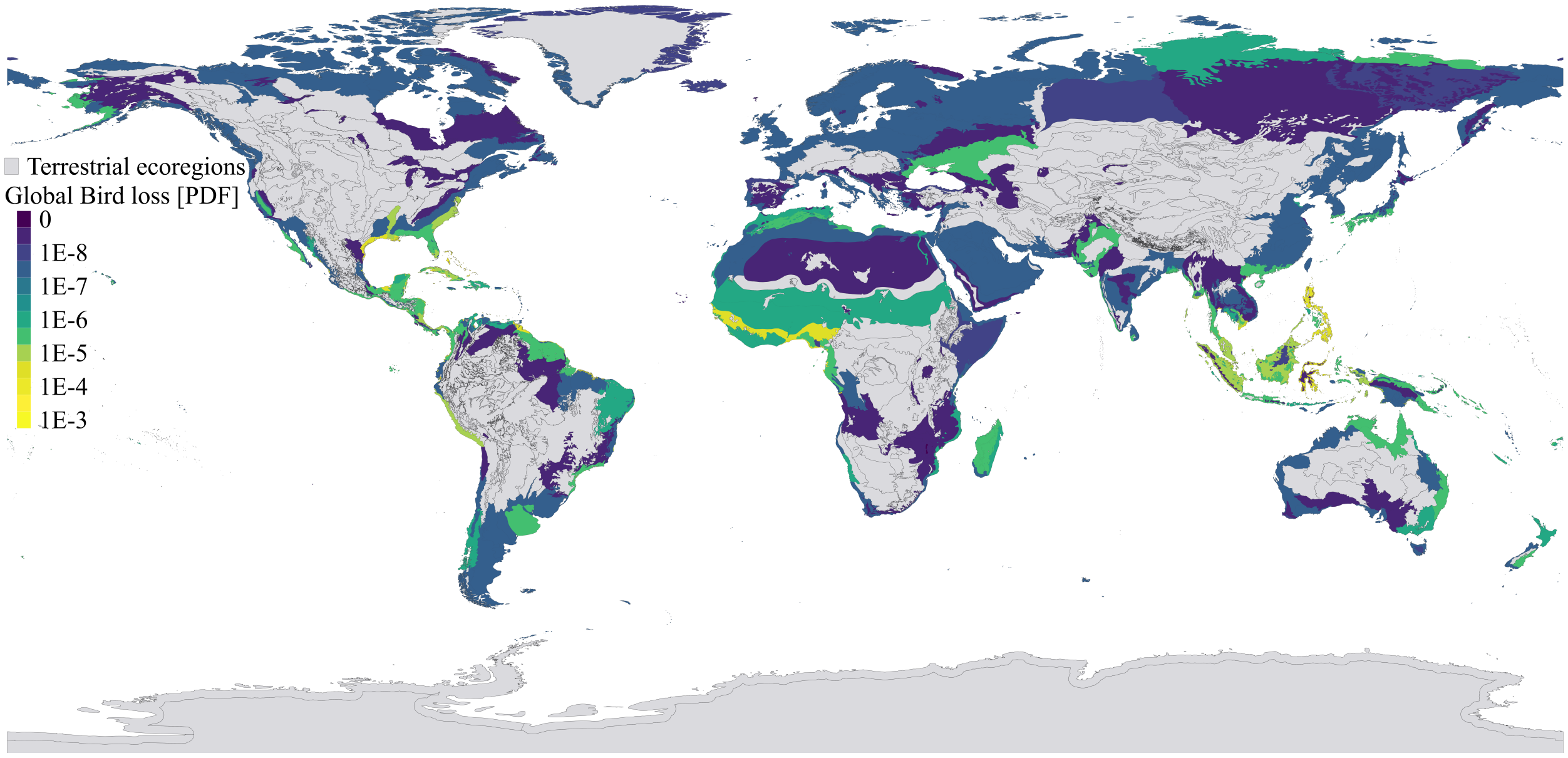

Figure S.11: Global species loss (SLR·EF·VF) by 2100 under SSP2 for birds [PDF]

Combined Characterization Factors

Table S7: Combined CFs for surface temperature increase[5] and SLR [PDF·yr/kg]

GHG	Combined CF Min	Combined CF SSP2	Combined CF Max
CO2	4.59E-17	4.61E-17	4.64E-17
CH4	1.74E-15	1.74E-15	1.74E-15
N2O	1.35E-14	1.35E-14	1.35E-14
CFC-11	2.52E-13	2.52E-13	2.53E-13
CFC-12	4.94E-13	4.95E-13	4.97E-13
CFC-13	5.865E-13	5.87E-13	5.89E-13
CFC-113	2.84E-13	2.84E-13	2.85E-13
CFC-114	3.93E-13	3.94E-13	3.95E-13
CFC-115	3.23E-13	3.24E-13	3.25E-13
HFC-125	1.87E-13	1.87E-13	1.88E-13
HFC-134a	7.87E-14	7.88E-14	7.90E-14
HFC-152a	1.05E-15	1.06E-15	1.09E-15
HFC-23	5.71E-13	5.72E-13	5.74E-13
HFC-32	3.82E-14	3.83E-14	3.84E-14
NF3	6.79E-13	6.81E-13	6.83E-13
PFC-116	4.66E-13	4.67E-13	4.68E-13
PFC-14	2.64E-13	2.64E-13	2.65E-13
SF6	9.96E-13	9.98E-13	1.00E-12
SF5CF3	7.45E-13	7.46E-13	7.48E-13
SO2F2	2.27E-13	2.28E-13	2.28E-13

References

(1) Zieger, V.; Lecompte, T.; Guihéneuf, S.; Guevel, Y.; Bazzana, M.; Gasser, T.; He, Y. Climate change metrics: IPCC AR6 updates, discussions and dynamic assessment applications. *EGUsphere* **2025**, 1–26.

(2) Scalgo SCALGO Compute. `https://scalgo.com/en-US/scalgo-compute`.

(3) Yamazaki, D.; Ikeshima, D.; Tawatari, R.; Yamaguchi, T.; O'Loughlin, F.; Neal, J. C.; Sampson, C. C.; Kanae, S.; Bates, P. D. A high-accuracy map of global terrain elevations. *Geophysical Research Letters* **2017**, *44*, 5844–5853.

(4) Kirezci, E.; Young, I. R.; Ranasinghe, R.; Muis, S.; Nicholls, R. J.; Lincke, D.; Hinkel, J.

Projections of global-scale extreme sea levels and resulting episodic coastal flooding over the 21st Century. *Scientific reports* **2020**, *10*, 1–12.

(5) Iordan, C.-M.; Kuipers, K. J. J.; Huang, B.; Hu, X.; Verones, F.; Cherubini, F. Spatially and taxonomically explicit characterisation factors for greenhouse gas emission impacts on biodiversity. *Resources, Conservation and Recycling* **2023**, *198*.

(6) Myhre, G.; Shindell, D.; Bréon, F.-M. F.-M.; Collins, W.; Fuglestvedt, J.; Huang, J.; Koch, D.; Lamarque, J.-F. J.-F.; Lee, D.; Mendoza, B.; Nakajima, T.; Robock, A.; Stephens, G.; Takemura, T.; Zhang, H. *Climate Change 2013: The Physical Science Basis. Contribution of Working Group I to the Fifth Assessment Report of the Intergovernmental Panel on Climate Change [Stocker, T.F., D. Qin, G.-K. Plattner, M. Tignor, S.K. Allen, J. Boschung, A. Nauels, Y. Xia, V. Bex and P.M. Midgley (eds.)]*; 2013; pp 1–44.

(7) Seeger, K.; Minderhoud, P. S. Sea level much higher than assumed in most coastal hazard assessments. *Nature* **2026**, 1–8.

(8) Scalgo About Us. `https://scalgo.com/en-US/about`.

(9) Scalgo SCALGO Live Documentation - Analysis - Sea-Level Rise. `https://scalgo.com/en-US/scalgo-live-documentation/analysis/sea-level-rise`.

(10) IPCC IPCC WGI Interactive Atlas. `https://interactive-atlas.ipcc.ch/`.

(11) Danish Agency for Climate Data The Danish Elevation Model (DK-DEM). Accessed 20.06.2025; `https://www.eng.klimadatastyrelsen.dk/green-transition-and-climate-adaptation/the-danish-elevation-model-dk-dem`.

(12) Pronk, M.; Hooijer, A.; Eilander, D.; Haag, A.; de Jong, T.; Vousdoukas, M.; Vernim-

men, R.; Ledoux, H.; Eleveld, M. DeltaDTM: A global coastal digital terrain model. *Scientific Data* **2024**, *11*, 273.

(13) Yamazaki, D.; Ikeshima, D.; Tawatari, R.; Yamaguchi, T.; O'Loughlin, F.; Neal, J. C.; Sampson, C. C.; Kanae, S.; Bates, P. D. MERIT DEM: Multi-Error-Removed Improved-Terrain DEM. http://hydro.iis.u-tokyo.ac.jp/~yamadai/MERIT_DEM/index.html.

(14) Li, J.; Wong, D. W. Effects of DEM sources on hydrologic applications. *Computers, Environment and urban systems* **2010**, *34*, 251–261.
